# TAGLN mediated stiffness-regulated ovarian cancer progression via RhoA/ROCK pathway

**DOI:** 10.1186/s13046-021-02091-6

**Published:** 2021-09-19

**Authors:** Xiao Wei, Hua Lou, Dongchen Zhou, Yijuan Jia, Huayi Li, Quanfu Huang, Jingjing Ma, Zongyuan Yang, Chaoyang Sun, Yunchong Meng, Sen Xu, Xin Yang, Xiaoting Li, Teng Ji, Zhongzhen Guo, Qinglei Gao

**Affiliations:** 1 Department of Obstetrics and Gynecology, Tongji Hospital, Tongji Medical College, Huazhong University of Science and Technology, 1095 Jiefang Anv, Wuhan, People’s Republic of China; 2 Department of Gynecology, The First Affiliated Hospital, Zhejiang University School of Medicine, Hangzhou, People’s Republic of China; 3 Department of Obstetrics and Gynecology, Wuhan First Hospital, Wuhan, People’s Republic of China; 4 Department of Thoracic Surgery, Wuhan Union Hospital, Tongji Medical College, Huazhong University of Science and Technology, Wuhan, People’s Republic of China; 5 Shenzhen Dapeng New District Maternity & Child Health Hospital Shenzhen Second People’s Hospital, The First Affiliated Hospital of Shenzhen University, Shenzhen, China

**Keywords:** Ovarian cancer, Progression, Tissue mechanics, TAGLN, RhoA/ROCK

## Abstract

**Background:**

Ovarian cancer (OC) progression is an unmet medical challenge. Since omental metastases were palpated harder than their primary counterparts during cytoreductive surgery of patients with epithelial ovarian cancer (EOC), we were inspired to investigate OC progression from the perspective of biomechanics.

**Methods:**

Atomic Force Microscope (AFM) was used to measure the Young’s modulus of tissues. The collagen-coated polyacrylamide hydrogel (PA gel) system was prepared to mimic the soft and stiff substrates *in vitro.* The effect of TAGLN was evaluated both *in vitro* and *in vivo* using transwell assay, immunofluorescence, western blot analysis and immunohistochemistry.

**Results:**

We quantitatively confirmed that omental metastases were stiffer and more abundant in desmoplasia compared with paired primary tumors, and further demonstrated that matrix stiffness could notably regulate OC progression. Remarkably, TAGLN, encoding an actin cross-linking/gelling protein, was identified as a potent mechanosensitive gene that could form a regulation loop with Src activation reacting to environmental stiffness, thus mediating stiffness-regulated OC progression through regulating RhoA/ROCK pathway.

**Conclusions:**

These data demonstrate that targeting extra-cellular matrix (ECM) stiffness could probably hamper OC progression, and of note, targeting TAGLN might provide promising clinical therapeutic value for OC therapy.

**Supplementary Information:**

The online version contains supplementary material available at 10.1186/s13046-021-02091-6.

## Background

Peritoneal metastasis is an important cause of high mortality of ovarian cancer (OC) [1], especially the metastasis to the omentum [2, 3], which results in transformation of this soft pad of tissue to a solid tumor [2]. Intriguingly, during ovarian cancer cytoreductive surgery, we noticed that these omental metastases are palpated significantly harder than their primary tumors of EOC. Since metastasis is a major difficulty of OC therapy [4] and metastases are palpated harder than primary tumors, we are interested in the correlation between ovarian cancer progression and tissue stiffness.

Tumors are often stiffer than normal tissues [5–7], frequently detected through physical palpation as a rigid mass residing within a compliant tissue [5]. This altered tissue-level and cellular mechanics is mainly due to extracellular matrix (ECM) remodeling [8, 9]. Recently, tumor mechanics have emerged as an important driving factor during tumor progression and chemotherapy sensitivity [10–12]. For example, in breast cancer, matrix stiffness drives epithelial–mesenchymal transition and tumor metastasis through a TWIST1–G3BP2 mechanotransduction pathway [10]. Given that, targeting ECM stiffness presents opportunities to attenuate tumor progression and improve the efficiency of chemotherapy [13, 14]. Cells perceive their microenvironment not only through soluble signals but also through physical and mechanical cues [15]. Past decades of research have mainly concentrated on the role of various extracellular and intracellular biochemical signals in tumor progression [16, 17], but whether and how mechanical cues play a role in the progression of OC and the underlying mechanism remain unclear.

Therefore, we aim to investigate whether and how tissue mechanics exerts influence on OC progression. We confirmed that omental metastases were stiffer than paired primary tumors and verified the impact of matrix stiffness on OC progression. Through transcriptomic array, TAGLN (transgelin) was identified as a mechanosensitive gene and formed a regulation loop with Src gene activation. We further demonstrated that TAGLN mediated stiffness-regulated OC progression through regulating RhoA/ROCK signaling pathway. Collectively, this study deepens our understanding of tumor biomechanics of OC progression and provides novel targets for OC therapy.

## Materials and methods

### Cell culture

The human ovarian cancer cell lines SK-OV-3 and ES-2 were purchased from American Type Culture Collection (ATCC) and were cultured in McCoy’s 5 A medium (Sigma-Aldrich) supplemented with 10 % fetal bovine serum (FBS) (Gibco) and 100 units/ml penicillin/streptomycin (Servicebio, Wuhan). All cells were maintained at 37 ℃ and 5 % CO_2_.

### Antibodies and reagents

The complete information on all the antibodies and reagents used in the present study is provided in supplementary Tables 1–2.

### Transfections

SiRNA transfections were performed using Lipofectamine 3000 transfection reagent (Thermo fisher scientific) according to the manufacturer’s instructions. siRNA of TAGLN (sequences: 5’-GGTTTATGAAGAAAGCGCAGGAGCA-3’), siRNA of YAP (sequences: 5’-CAGCAGAAUAUGAUGAACUCGGCUU-3’) and negative control siRNA (siControl) were ordered from Invitrogen (Thermo Fisher Scientific, Inc). Constitutively active Src pLNCX chick src Y416F was from Addgene (plasmid#13,662). TAGLN expression plasmid (CH841680) and vector plasmid (PD88001) were purchased from Vigene (Biosciences). Transfection of plasmid was performed using X-tremeGENE HP DNA Transfection Reagent (Roche) according to the manufacturer’s instructions. Cells were transfected with hU6-MCS-Ubiquitin-firefly_Luciferase-IRES-puromycin lentiviral particles, constructed by GeneChem Co., Ltd (Shanghai, China), to knockdown the expression of TAGLN in tumor cells. The sequence targeted by sh-TAGLN was: 5’-ATG TCA TTG GCC TTC AGA T-3’.

### Immunofluorescence and imaging

Cells were fixed with 4 % paraformaldehyde for 30 min at room temperature (RT) and permeabilized and blocked with 0.1 % Triton X-100 in 5 % BSA for 20 min at RT. All samples were incubated with primary antibody at 4℃ overnight and followed by Alexa-conjugated secondary antibody for one hour at RT. Cells were subsequently stained with 10 µM phalloidin-TRITC (Sigma-Aldrich) for 30 min. Nuclei were counterstained with diaminophenylindole (DAPI) (Sigma-Aldrich). Immunofluorescence staining was visualized using fluorescence microscopy (Olympus DP73, Tokyo, Japan).

### Clinical samples

Human ovarian cancer tissues were all obtained from patients undergoing surgery at the Department of Obstetrics and Gynecology, Tongji Hospital, Huazhong University of Science and Technology after obtaining written informed consent of the patients and the authorization of the Ethics Committee of Tongji Hospital (TJ-iRB20181103). Fresh tissues were prepared for the Atomic Force Microscope (AFM) measurement, frozen or formalin fixed.

For Kaplan-Meier analysis, TAGLN expression was detected in our samples of 132 OC patients on the available clinical data including time of diagnosis, death, or last follow-up. Progression-free survival (PFS) was calculated based on the date of initial surgery to the date of progression/recurrence or last known contact. The cut-off for “high” and “low” TAGLN is the mean value of TAGLN expression score. Overall survival (OS) was calculated based on the date of initial surgery to death or last known contact. The status of PFS and OS regarding TAGLN expression was estimated with the Kaplan-Meier methods using GraphPad Prism 6 software, and the log-rank test was used to evaluate the statistical significance. Patient characteristics are summarized in Supplementary Tables 4, 5, 6, 7 and 8.

### Preparation of polyacrylamide (PA) gels substrates

The preparation of polyacrylamide (PA) gels substrates was described previously [18]. The Young’s modulus of PA gel substrates were measured using an atomic force microscopy (SPM9700 scanning probe microscope, Shimadzu).

### Tissue preparation for AFM measurements of ECM stiffness

Tissues for AFM measurement were prepared as Plodinec et al. described [19]. Both human ovarian cancer tissues and tumors removed from xenograft models were immediately immersed in cold PBS and kept at 4 °C until an experiment commences. These tissues could be kept up for three days before the AFM measurement. Dissect the sample into 1–3 mm thick slices as parallel and smooth as possible for high quality measurements. To attach the sample to a hard substrate during the measurement, we used two-component epoxy glue (5 min hardening time). Apply a thin layer of mixed epoxy glue onto the glass of 12 mm diameter and dry briefly the bottom side using a lint-free tissue paper, then attach the sample onto the glue. After the epoxy was hard enough and the tissue sample was stable attached, we transferred the tissue-attached glass to conduct the AFM measurement.

### Atomic force microscopy measurements

All AFM measurements were performed using an atomic force microscope (SPM9700 scanning probe microscope, SHIMADZU, Japan) in Huazhong University of Science and Technology. The AFM measurements were conducted as previously described [18]. Ten different positions per tissues were measured and averaged.

### Animal studies

Female BALB/c-nu mice (5 weeks old) were purchased from the Animal Experimental Center (Shanghai, China). All animal experiments were performed with the approval of the Committee on the Ethics of Animal Experiments in Hubei Province (Institutional Review Board ID: TJ-A20160102). All mice were housed under pathogen-free conditions in the Animal Research Center of Tongji Hospital at 22 °C with 12-hour light/dark cycles and free access to water and food. An orthotopic model of ovarian cancer was established as we described previously [20]. Briefly, 2 × 10^6^ cells in 25 µL PBS were injected orthotopically under the ovarian bursa. Seven days after cells injection, mice were randomly assigned to treatment groups to avoid treatment bias. For BAPN treatment, BAPN was given i.p. (50 mg/kg) every 2 days for 4 weeks, or saline was used as a control. For dasatinib treatment, dasatinib was given i.p. (15 mg/kg) every 2 days for 4 weeks, or DMSO was used as a control. For TAGLN *in vivo* experiment, 2 × 10^6^ SK-OV-3 cells ectopically knockdown of TAGLN (sh-TAGLN, *n* = 6 per group), or respective appropriate control (sh-Control, *n* = 6 per group) were injected orthotopically under the ovarian bursa BALB/c-nu mice. After weeks of injection, the mice were anaesthetized with 1 % pentobarbital sodium, and imaged with the IVIS SPECTRUM system (Caliper, Xenogen, USA). Total flux (photons/s) of xenografts was analyzed utilizing Living Image version 4·3·1 software. After executing the mice, tumors were resected, prepared for AFM measurement, frozen or fixed with paraformaldehyde.

### Statistical analysis

All data were presented as the mean ± SEM. Two experiments groups were compared by using a two-tailed paired *t*-test for paired data or a two-tailed unpaired *t*-test for unpaired data after confirming the normality of the data. Analysis of variance was used to compare differences among multiple groups (post hoc test: Turkey’s multiple comparisons test). Pearson correlation was used to analyze a potential correlation between two different experimental parameters. GraphPad Prism 6.0 software was used to conduct the statistical analysis of the data. *P* values less than or equal to 0.05 were considered to be significant.

### Cell track

Cells were cultured on soft and stiff PA gels (at six-wells) at a density of 8000 cells per well for over 24 h. Cell movement was recorded using live-cell imaging (laser scanning confocal microscopy, IX83 living cell imaging system, Olympus FV1000, Tokyo, Japan) at 6-min intervals over a 16-h period and multiple position imaging was gathered. At least three independent experiments were performed; 20–40 cells were analyzed in each experiment. Cell movement was analyzed using Chemotaxis and Migration Tool plug-in (Ibidi) of ImageJ software. Total length of the cell track (in µm) was generated for multiple cells, averaged and normalized to cell tracks from cells cultured on soft PA gels.

### Other methods

Masson trichrome and picrosirius red staining, Immunohistochemistry, Quantitative real-time PCR, Western blot analysis, Transwell assay, Rho GTPase activity, Gene set enrichment analysis and transcriptomic array were in supplementary methods.

## Results

### Ovarian cancer metastases exert higher stiffness than primary tumors

**Fig. 1:**
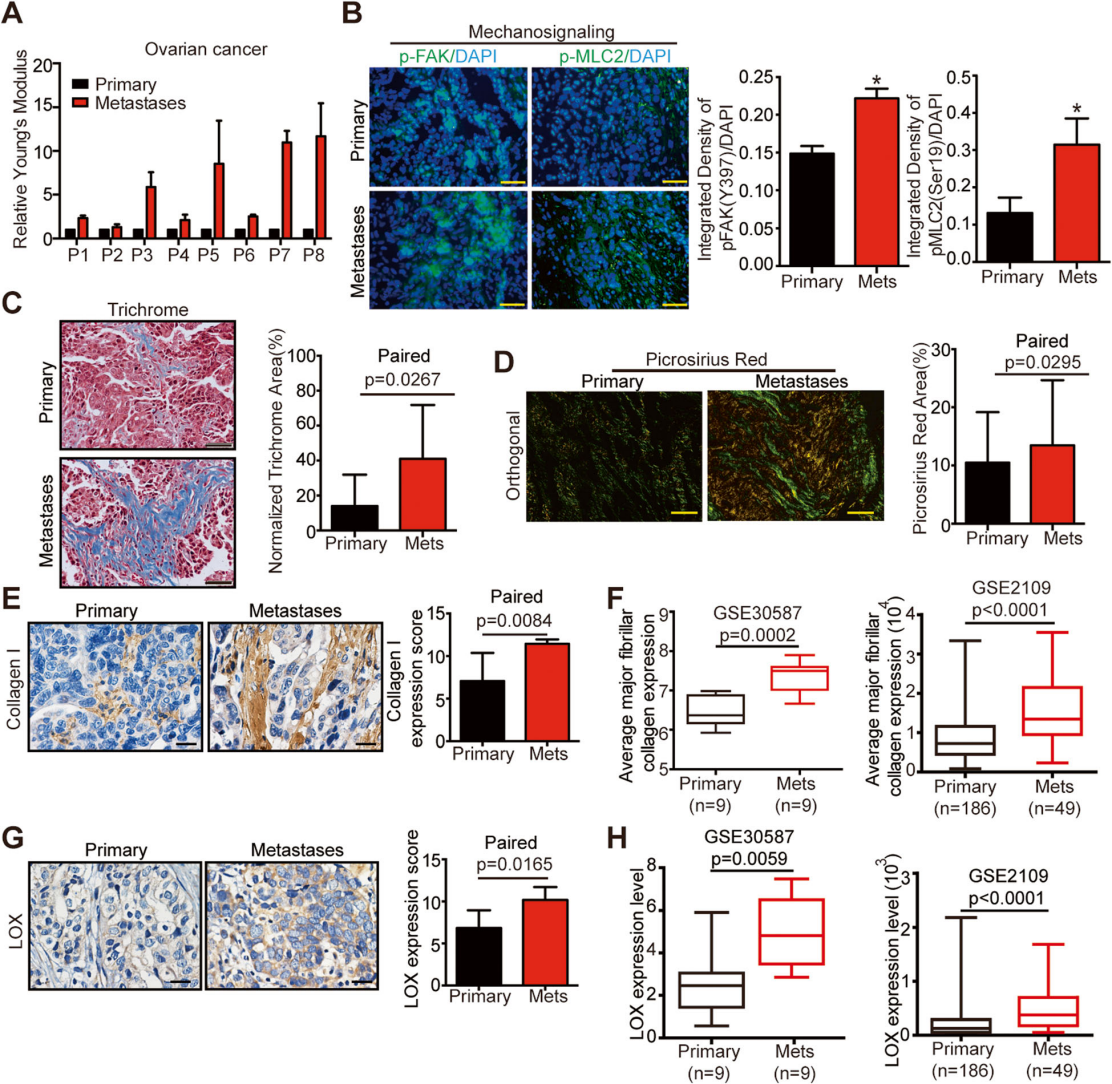
Ovarian cancer metastases exert higher stiffness than primary tumors. **A** The Young’s modulus of paired primary tumors and omental metastases of HGSOC assessed by AFM analysis. Data are relative average compressive Young’s modulus, analyzed from eight paired samples per condition. **B** Representative immunofluorescence images (left) and quantification (right) for p-FAK (Y397) (green, left) and p-MLC2 (green, middle) with DAPI (blue) for paired tumors of HGSOC (*n* = 3, *P < 0.05). Scale bar: 50 μm. **C** Trichrome staining (left) of paired tumors of HGSOC and quantification (right) (*n* = 8). Scale bar: 50 μm. **D** Photomicrographs of paired tumors of HGSOC in stained with picrosirius red, viewed under orthogonal polarizing filters (left) and quantification (right) (*n* = 8). Scale bar: 50 μm. **E** Immunohistochemistry (IHC) assess of collagen I in paired tumors of HGSOC (left: representative images of IHC staining, right: quantification of collagen I expression, *n* = 8). Scale bar: 20 μm. **F** Boxplots showing the expression level of average major fibrillar collagen signature in paired (left) or unpaired (right) primary tumors and metastases of ovarian cancer included in ovarian dataset. **G** Immunohistochemistry (IHC) assess of LOX in paired tumors of HGSOC (left: representative images of IHC staining, right: quantification of LOX expression, *n* = 8). Scale bar: 20 μm. **H** Boxplots showing the expression level of LOX in paired (left) or unpaired (right) primary tumors and metastases of ovarian cancer included in ovarian dataset

The surgeons frequently found that the peritoneal metastases of OC harbored higher stiffness than their primary counterparts. To confirm it, we utilized atom force microscope (AFM) to quantify the stiffness of eight paired primary tumors and omental metastases of EOC, showing that the Young’s modulus of metastases were higher than that of paired primary tumors [Fig. 1A]. Besides, increased mechanosignaling expressions including phosphorylated focal adhesion kinase (pFAK residue Tyr397) and myosin light chain 2 (pMLC2 residue Ser19) were detected in metastases when compared with primary tumors [Fig. 1B].

ECM deposition and collagen crosslinking are proven to contribute to increased tissue stiffness [9]. As revealed by trichrome and picrosirius staining, the stroma of metastases contained more fibrillar collagen than primary tumors [Fig. 1C and D]. Besides, higher expression of collagen Ι was observed in metastases than in primary tumors [Fig. 1E]. Moreover, bioinformatics analysis of paired and unpaired public gene data (GSE30587 and GSE2109) confirmed the elevated expression of major fibrillar collagens signature [21] in the metastases [Fig. 1F and Supplementary Table 3]. Lysyl oxidase (LOX), a key enzyme that initiates the crosslink of collagen, was more abundantly expressed in the metastases than in primary tumors [Fig. 1G], which was confirmed by the analysis of public gene data [Fig. 1H]. To conclude, these data demonstrated that the metastases of EOC were stiffer than primary tumors, and the increased stiffness could be attributed to the upregulation of desmoplasia in the metastases.

### Matrix stiffness modulates ovarian cancer progression and activates Src gene and RhoA/ROCK pathway

**Fig. 2:**
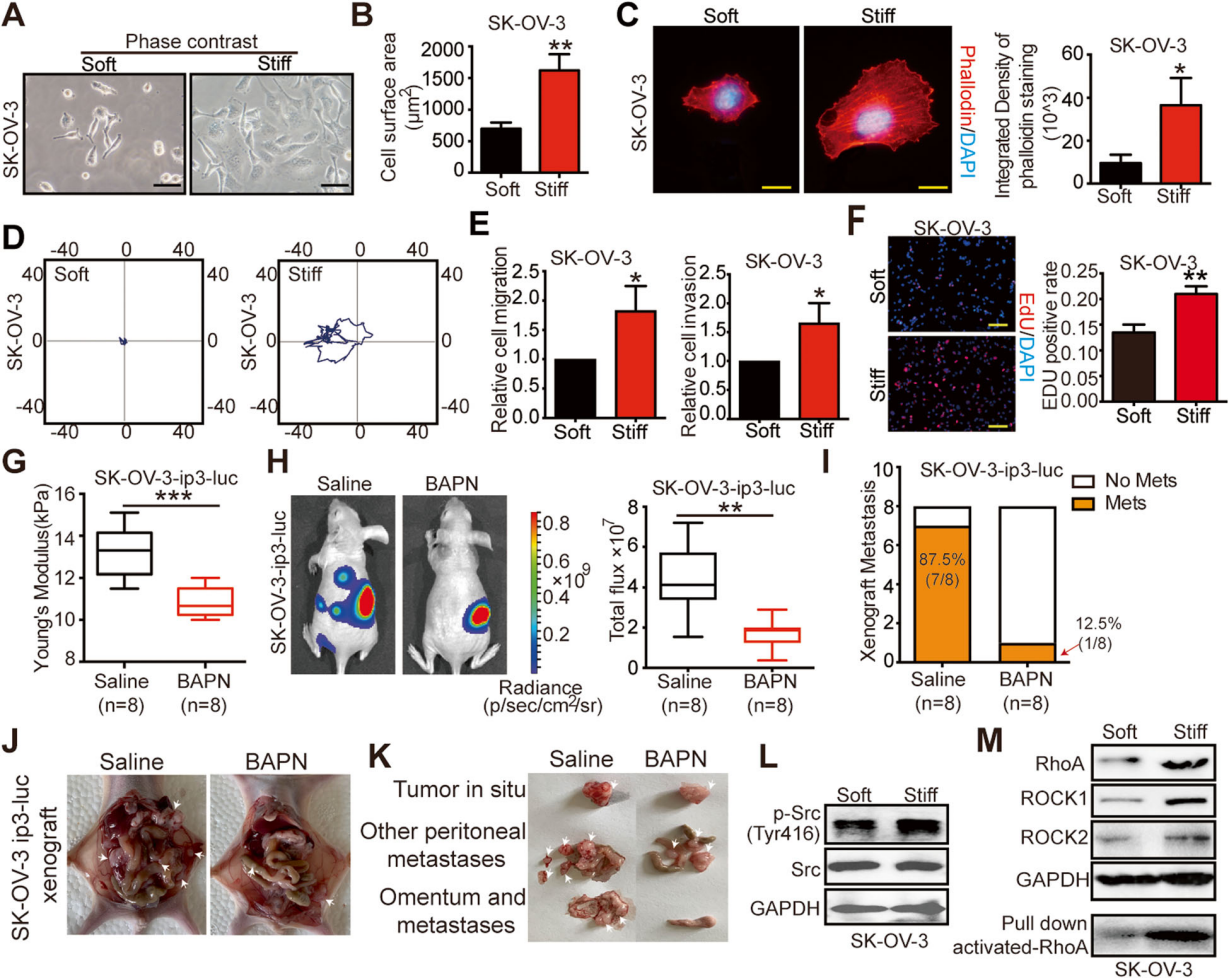
Matrix stiffness modulates ovarian cancer progression and activates Src gene and RhoA/ROCK pathway. **A** Phase images showing typical morphology of SK-OV-3 cells cultured on soft and stiff collagen I coated PA gels. Scale bar: 50 μm. **B** Cell surface areas calculated by digital image analysis of phase-contrast images of cells on soft and stiff collagen I coated PA gels (**P < 0.01). **C** Representative immunofluorescence images of SK-OV-3 cells (left) and quantifications (right) for phalloidin (red) and with DAPI (blue) (*P < 0.05). Scale bar: 20 μm. **D** Migratory tracks of SK-OV-3 cells cultured on soft or stiff substrates. **E** Migratory (left) and invasive (right) ability of SK-OV-3 cells cultured on soft versus stiff substrates evaluated by transwell assays (*P < 0.05). **F** The proliferation of SK-OV-3 cells cultured on soft versus stiff substrates was measured by EdU assay, DAPI (blue). Scale bar: 50 μm. (right) Quantification of EdU-positive nuclei from *n* = 3 experiments (**P < 0.01). **G** Young’s modulus of tumors from mice treated with saline or BAPN (*n* = 8 each group) were assessed by AFM (***P < 0.001). **H** Representative bioluminescence images of mice (*n* = 8 each group) treated with saline or BAPN for 4 weeks after tumor implantation. Bar graph showing the quantification of normalized total photon counts in each group (*n* = 8 each group, **P < 0.01). **I** Incidence of metastases was decreased by BAPN treatment. In saline-treated group, seven mice have peritoneal metastasis, the incidence of metastasis was 87.5 %; in BAPN-treated mice, only one mouse has peritoneal metastasis, the incidence of metastasis was 12.5 % (*n* = 8). **J-K** Representative macroscopic images (**J**) of SK-OV-3-ip3-luc orthotopic xenograft treated with saline or BAPN. **K** Tumors from each group were resected and counted. **L** Western blot from cell lysates of SK-OV-3 cells showing expression of p-Src (Try416), Src and GAPDH. GAPDH was used as a loading control. **M** Western blot from cell lysates of SK-OV-3 cells on soft and stiff substrates showing expression of, RhoA, ROCK1, ROCK2 and GAPDH. GAPDH was used as a loading control. Activated-RhoA was detected by RhoA pull down analysis

Tumor cells from the metastases were reported to have more aggressive biology than those from the primary sites [4]. To explore whether matrix stiffness could modulate EOC progression, the collagen-coated polyacrylamide hydrogel (PA gel) system with calibrated Young’s modulus of 0.25 ± 0.05 kPa and 10.5 ± 0.02 kPa was prepared to mimic the soft and stiff substrates *in vitro *[22]. Both SK-OV-3 and ES-2 cells exhibited well-spread morphology [Fig. 2 A, supplementary Fig. 1A] and significantly larger surface areas in response to stiff substrates [Fig. 2B, supplementary Fig. 1B]. Additionally, increased cytoskeleton assembly was observed in cells cultured on stiff substrates [Fig. 2 C, supplementary Fig. 1C], implying that stiffness might affect cell motility. Compared with cells on soft substrates, those cultured on stiff ones showed augmented migratory, invasive, and proliferative capabilities [Fig. 2D–F and Supplementary Fig. 1D and E], indicating that matrix stiffness could impose influences on malignant behaviors of EOC cells.

To further investigate the effects of matrix stiffness on OC progression *in vivo*, we conducted an orthotopic OC xenograft and used β-aminopropionitrile (BAPN), a classical LOX inhibitor that was commonly used in interrogating mechanical properties of ECM [13], to decrease the tumor stiffness *in vivo*. The administration of BAPN significantly downregulated matrix stiffness [Fig. 2G] and attenuated the tumor burden in mice when compared with controls using saline [Fig. 2 H]. Moreover, tumors from BAPN-treated mice displayed weakened expression of Ki67 [supplementary Fig. 2], indicating that decreased matrix stiffness sustained the proliferation of OC *in vivo*. Importantly, the BAPN-treated mice had lower incidence of peritoneal metastasis (12.5 %), compared with saline-treated group (87.5 %) [Fig. 2I] and less frequently occurred, smaller secondary loci [Fig. 2 J and K] than saline-treated controls. Taken together, our *in vitro* and *in vivo* data demonstrated that matrix stiffness could modulate OC progression.

By activating mechanosignaling molecules such as Src family kinases [23], cells perceive and respond to physical signals, thereby transforming their biological behaviors [9]. Significantly, higher expressions of p-Src^Try416^ were observed in OC cells cultured on stiff substrates [Fig. 2 L, supplementary Fig. 3A]. Consistently, tumors of mouse models that received BAPN displayed decreased expression of p-Src^Try416^ [supplementary Fig. 3B]. Rho (Ras homology) GTPases family members and the downstream factor ROCK has been demonstrated important in cell migration and mechanotransduction process [24]. Markedly, OC cells cultured on stiff substrates harbored higher expressions of RhoA, particularly activated RhoA, and downstream ROCK1 and ROCK2 compared with cells cultured on soft substrates [Fig. 2 M, supplementary Fig. 3C]. Similar results were observed in BAPN-treated xenograft experiments [supplementary Fig. 3D-E]. Together, these data showed that Src gene and RhoA/ROCK mechanosignaling pathway were activated in OC in response to mechanical cues.

### TAGLN is identified as a stiffness-regulated gene in ovarian cancer

**Fig. 3:**
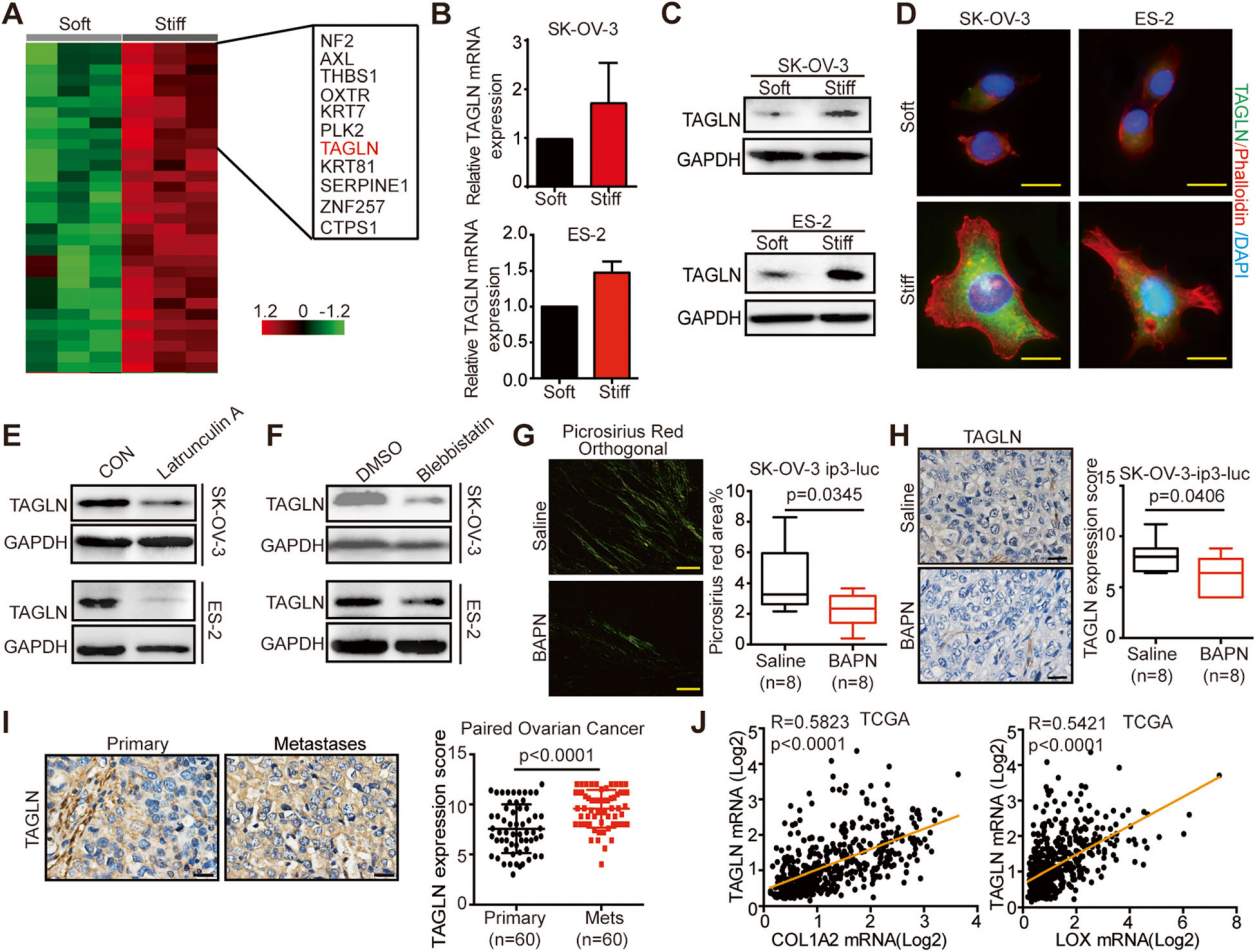
TAGLN is identified as a stiffness-regulated gene in ovarian cancer. **A** Transcriptomic profiling of SK-OV-3 cells cultured on soft (0.25 kPa) or stiff (10.5 kPa) PA gels. The heat map shows genes upregulated in SK-OV-3 cells cultured on stiff PA gels compared to soft PA gels. Green or red in the heat map indicate genes expression that was relatively low or high, respectively. **B** Relative TAGLN mRNA expression levels of SK-OV-3 and ES-2 cells cultured on soft versus stiff substrates determined by quantitative PCR. **C** Western blot determined the expression of TAGLN in SK-OV-3 and ES-2 cells cultured on soft or stiff substrates. **D** Immunofluorescence of SK-OV-3 and ES-2 cells cultured on soft or stiff substrates: TAGLN (green), phalloidin (red), and DAPI (blue). Scale bar: 20 μm. **E** Western blot showing TAGLN expression in SK-OV-3 and ES-2 cells treated with Latrunculin A (0.1 µM, 24 h). GAPDH was used as a loading control. **F** Western blot showing TAGLN expression in SK-OV-3 and ES-2 cells treated with blebbistatin (10 µM, 24 h). GAPDH was used as a loading control. **G** Photomicrographs of paired tumors of HGSOC in stained with picrosirius red, viewed under orthogonal polarizing filters (left) and quantification (right) (*n* = 8). Scale bar: 50 μm. **H** Representative images (left) and scores (right) of IHC staining for TAGLN from mice treated with saline or BAPN (*n* = 8). Scale bar: 20 μm. **I** IHC staining for TAGLN expression of tissues from paired tumors of HGSOC. Representative images were shown left (Scale bar: 20 μm) and scores right (*n* = 60). **J** Pearson correlation analysis of TAGLN with COL1A2 and LOX in TCGA datasets

To investigate the molecular mechanisms that OC cells respond to the mechanical cues, we performed transcriptomic microarray analysis of SK-OV-3 cells cultured on soft and stiff substrates. Gene set enrichment analysis (GSEA) indicated the enrichment of “CYTOSKELETON” and “ACTIN_FILAMENT-BINDING” gene sets in cells cultured on stiff substrates [supplementary Fig. 4], further demonstrated the impact of tissue stiffness on cytoskeleton, as we showed in Fig. 2B. Cytoskeleton alternation is an important cellular response to matrix stiffness [8]. Within the pool of differentially expressed genes, TAGLN, an actin cross-linking/gelling protein sensitive to morphologic alternations, was drastically upregulated upon mechanical stimulation [Fig. 3 A], even most significantly upregulated when cells were cultured on substrates with greater stiffness (40 kPa) [supplementary Fig. 5A]. Significantly, higher TAGLN expression was detected in OC cells cultured on stiff substrates, compared with cells cultured on soft substrates both at the mRNA level [Fig. 3B, supplementary Fig. 5B] and the protein level [Fig. 3C-D]. We also detected higher expression of other upregulated genes, such as THBS1, OXTR, in cells cultured on stiff substrates [supplementary Fig. 5C], but they did not show association with cytoskeleton, thus, we identified TAGLN to continue the study. To further elucidate the mechanosensitivity of TAGLN, we treated OC cells with latrunculin A, an inhibitor of actin assembly and polymerization, which resulted in notably weakened TAGLN expression [Fig. 3E]. The inhibition of non-muscle myosin with Blebbistatin could also downregulate the expression level of TAGLN [Fig. 3 F]. Besides inhibition of the crosslink of collagen, BAPN could also decrease expression of fibrillar collagen [Fig. 3G]. For the *in vivo* experiment, tumors of BAPN-treated mouse models, which has been proved softer, showed significantly less expression of TAGLN than saline-group [Fig. 3H].

In addition, TAGLN expression of omental metastases of OC patients was significantly greater than that of paired primary tumors [Fig. 3I], which was also verified using public paired (GSE30587) and unpaired (GSE2109) profiling data [Supplementary Fig. 6]. Moreover, analysis of TCGA database indicated that the expression of TAGLN positively correlated with that of COL1A2 and LOX, two pivotal genes involved in matrix stiffness [Fig. 3 J]. To summarize, these results unraveled that TAGLN was a stiffness-regulated gene in OC that might play an important role in stiffness-related OC progression.

### TAGLN mediates stiffness-regulated ovarian cancer progression and correlates with poor patient prognosis

**Fig. 4:**
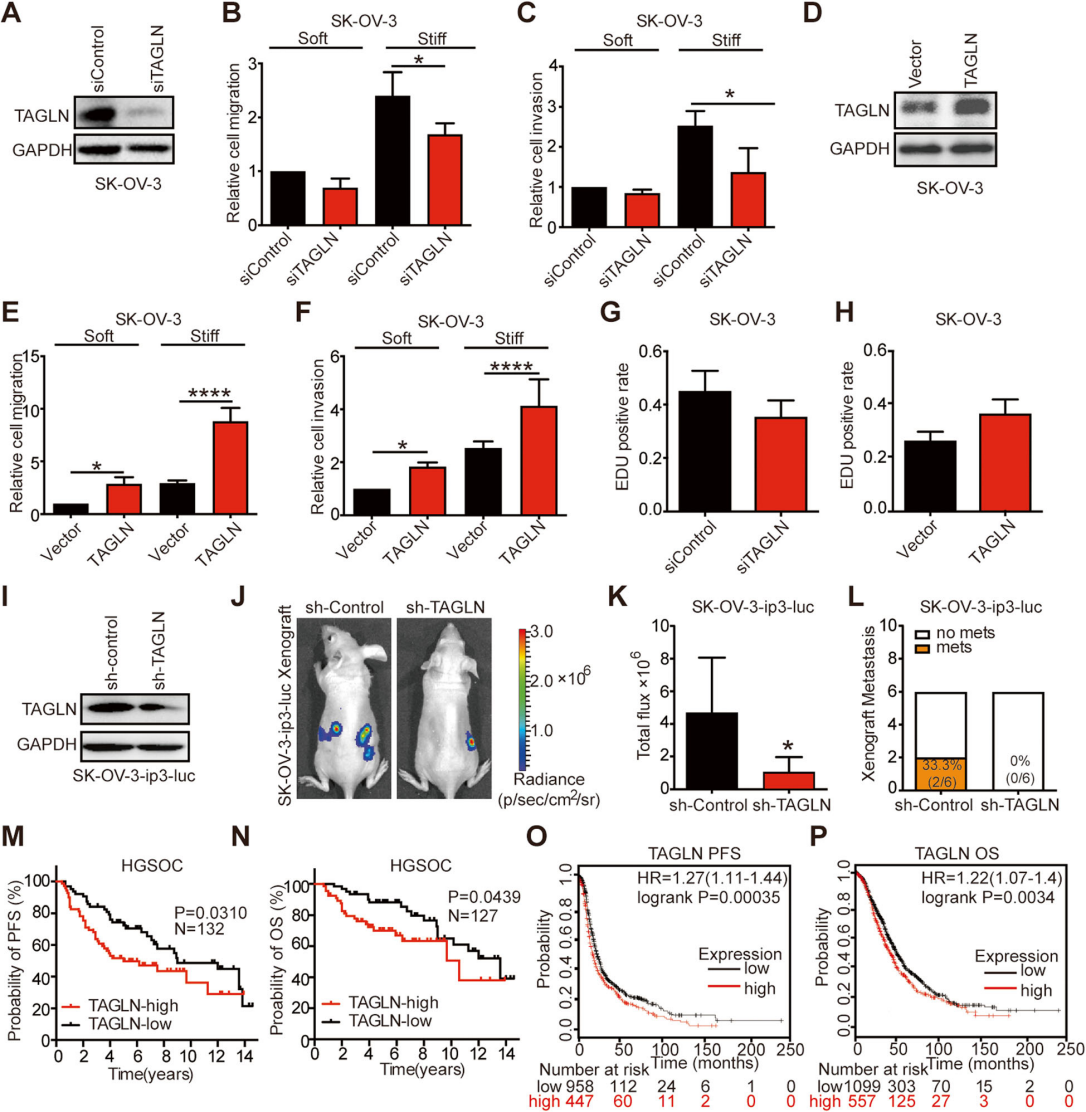
TAGLN mediates stiffness-regulated ovarian cancer progression and correlates with poor patient prognosis. **A** Knockdown of TAGLN by siRNA in SK-OV-3 cells were determined by western blot and probed for TAGLN and GAPDH. **B-C** Migratory (**B**) and invasive (**C**) ability of SK-OV-3 cells transfected with siControl or TAGLN siRNA, cultured on soft or stiff substrates, were evaluated by transwell assays and quantified (*P < 0.05). (**D**) Overexpression of TAGLN by plasmid transfection in SK-OV-3 cells were determined by western blot and probed for TAGLN and GAPDH. **E-F** Migratory (**E**) and invasive (**F**) ability of SK-OV-3 cells with the expression of control vector or overexpression of TAGLN protein, cultured on soft or stiff substrates, were evaluated by transwell assays and quantified (*P < 0.05, ****P < 0.0001). **G-H** The proliferation of SK-OV-3 cells down-regulated (**G**) or overexpressed TAGLN (**H**) was measured by EdU assay. Quantification of EdU-positive nuclei from *n* = 3 experiments. **I** Cell lysates from SK-OV-3-ip3-luc transfected with lentivirus of sh-control and sh-TAGLN were determined by western blot and probed for TAGLN and GAPDH. **J-K** Representative bioluminescence images (**J**) of mice bearing SK-OV-3-ip3-luc cells transfected with sh-control or sh-TAGLN at 4 weeks after tumor implantation. Bar graph (**K**) showing the quantification of normalized total photon counts in each group (*n* = 6, *P < 0.05). **L** Incidence of metastases was decreased in sh-TAGLN group (*n* = 6 each group). In sh-control group, two mice have peritoneal metastasis, the incidence of metastasis was 33.3 %; in sh-TAGLN group, no mouse has peritoneal metastasis, the incidence of metastasis was 0 %. **M-N** Kaplan–Meier analysis of PFS (**M**) and OS (**N**) of ovarian cancer patients classified by tumor TAGLN protein levels into high and low expression group. **O-P** PFS (**O**) and OS (**P**) in ovarian cancer patients with low and high levels of TAGLN (Affymetrix probeset 205547_s_at). PFS: progression-free survival, OS: overall survival

To determine the impact of TAGLN in stiffness-regulated OC progression, we first successfully established SK-OV-3 and ES-2 cells deficient in TAGLN expression using siRNA-based approach [Fig. 4A and supplementary Fig. 7A]. The knockdown of TAGLN expression impaired both migratory and invasive capabilities of cells, especially for cells cultured on stiff substrates [Fig. 4B-C, supplementary Fig. 7B-C]. Furthermore, upregulating TAGLN levels using an expression plasmid [Fig. 4D, supplementary Fig. 7D] enhanced the capabilities of migration and invasion of both OC cells cultured on soft and stiff substrates [Fig. 4E-F, supplementary Fig. 7E-F]. Besides, cell proliferation was higher when TAGLN was upregulated [Fig. 4G] and lower when TAGLN was downregulated in [Fig. 4H] OC cells.

To further investigate the role of TAGLN in OC progression *in vivo*, we generated an orthotropic OC xenograft by injection of SK-OV-3-ip3-luc cells, in which TAGLN expression was downregulated through lentivirus transfection [Fig. 4I]. We observed that silencing TAGLN using shRNA restrained tumor progression [Fig. 4J and K] and lowered the metastasis rate [Fig. 4L], suggesting that TAGLN mediated stiffness-regulated OC progression.

Given the importance of TAGLN in mediating stiffness-regulated OC progression, we explored whether TAGLN expression correlated with OC prognosis. Analysis of pan-cancer Genomic Profiles indicated that increased TAGLN was associated with poor survival in most epithelial cancers [25], especially OC [supplementary Fig. 8A]. Besides, among 132 patients with EOC, we found that high level of TAGLN protein (defined by IHC) was significantly associated with both shortened PFS and OS [Fig. 4 M and N]. The Kaplan-Meier survival analysis also indicated that high expression of TAGLN was correlated with poorer PFS [HR = 1.27 (95 % CI 1.11–1.44) Fig. 4O] and OS [HR = 1.22 (95 % CI 1.07–1.40) Fig. 4P] in our patient cohort and curatedOvarianData [26] [supplementary Fig. 8B and C]. Thus, we confirmed the association between high TAGLN expression and poor prognosis of OC.

**Fig. 5:**
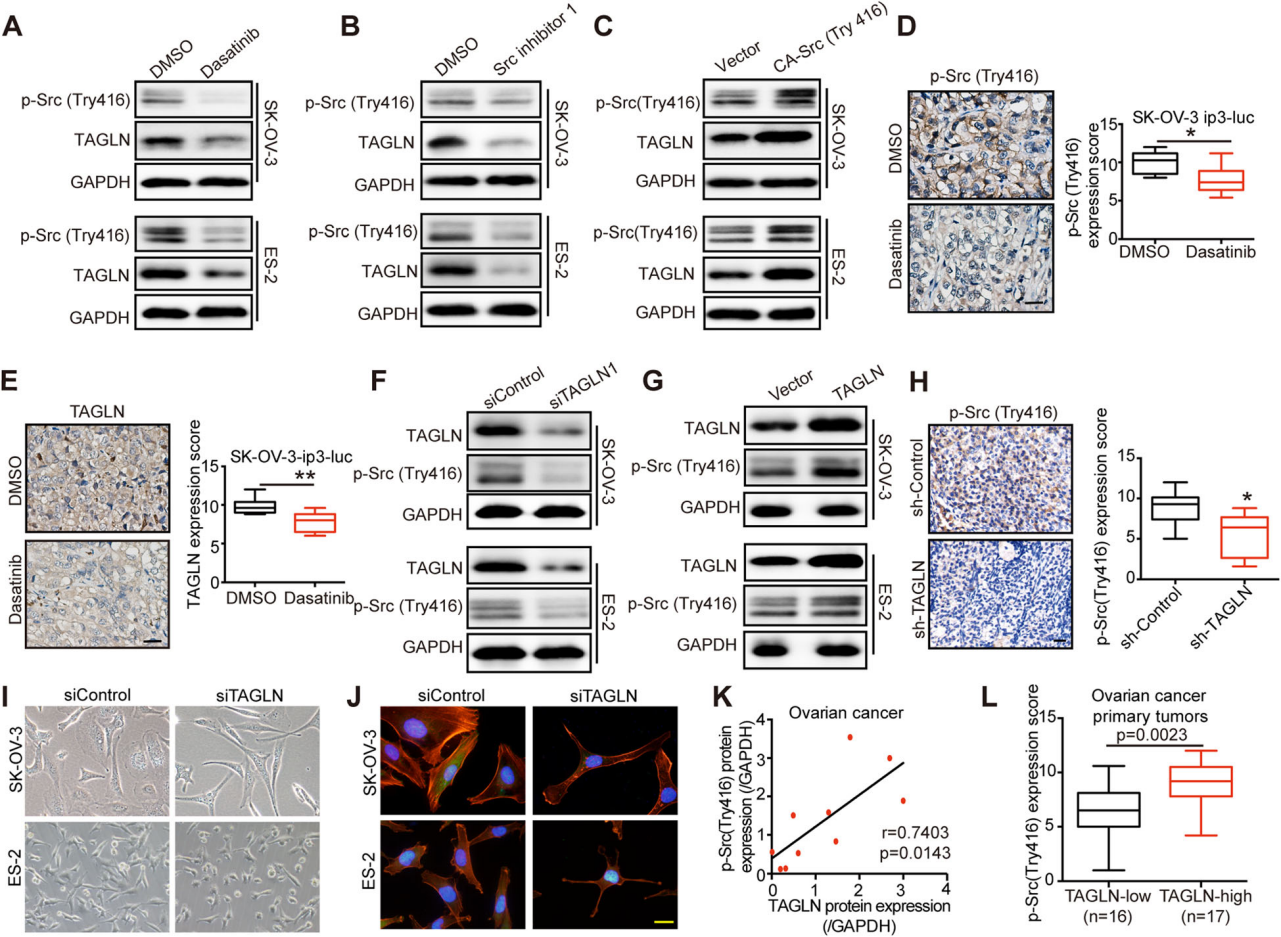
TAGLN and Src activation form a regulation loop. **A** Western blot analyzed TAGLN and p-Src (Try416) expression of cells treated with dasatinib (20 nM, 24 h). GAPDH was used as a loading control. **B** Western blot analyzed TAGLN and p-Src (Try416) expression of cells treated with Src inhibitor 1 (50 nM, 24 h). GAPDH was used as a loading control. **C** Lysates from cells transfected with vector or active Src Y416F plasmid (CA-Src416) were analyzed by SDS–PAGE and probed for TAGLN, p-Src (Try416) and GAPDH. **D-E** Representative images of IHC staining and scores of p-Src (Try416) (**D**)and TAGLN (**E**) from mice treated with DMSO or dasatinib (*n* = 8, *P < 0.05, **P < 0.01). Scale bar: 20 μm. **F** Western blot analyzed TAGLN and p-Src (Try416) expression of cells transfected with siControl or siTAGLN. GAPDH was used as a loading control. **G**Western blot analyzed TAGLN and p-Src (Try416) expression of cells transfected with vector or TAGLN plasmid. GAPDH was used as a loading control. **H** Representative images (left) and scores (right) of p-Src (Try416) IHC staining of tumor tissues from mice bearing SK-OV-3 cells transfected with sh-control or sh-TAGLN (*P < 0.05). Scale bar: 20 μm. **I** Phase images showing typical morphology of SK-OV-3 and ES-2 cells transfected with siControl or siTAGLN. **J** Representative immunofluorescence images of SK-OV-3 and ES-2 cells transfected with siControl or siTAGLN for phalloidin (red) and with DAPI (blue). Scale bar: 20 μm. **K** Correlation between TAGLN and p-Src (Try416) protein expression in primary tumors of ovarian cancer tissues (*n* = 10 biological replicate samples). **L** Immunohistochemical analysis of TAGLN and p-Src (Try416) expression from primary tumors of EOC. The scores of p-Src (Try416) in the TAGLN-low group (*n*=16) and TAGLN-high group (*n*=17) was calculated

### TAGLN and Src activation form a regulation loop

Given that the activation of Src was pivotal in mechanotransduction of OC cells [27], we hypothesized that Src modulated the expression of TAGLN. To investigate whether alternations of Src activation could impose influences on the expression level of TAGLN, we treated cells with Src inhibitors (dasatinib or Src inhibitor 1) or constitutively active Src [CA-Src (Try416)]. Intriguingly, we found that inhibition of Src evidently suppressed the expression of TAGLN [Fig. 5A and B]. Overexpression of constitutively active Src (CA-Src (Try416) with plasmid markedly upregulated TAGLN expression [Fig. 5 C]. To further illustrate the effect of Src activation on TAGLN expression *in vivo*, we treated OC xenograft with dasatinib. Compared with those in DMSO-treated group, the expression level of TAGLN and phosphorylation of Src in mice treated with dasatinib were significantly curtailed [Fig. 5D-E], indicating that Src activation regulated TAGLN expression *in vivo*. To decipher how Src activation affects TAGLN expression, we investigated YAP, another key component of mechanosignaling pathway [15]. The inhibition of Src activation using dasatinib or Src inhibitor 1 could effectively suppress YAP expression [supplementary Fig. 9 A-B], moreover, silencing YAP could significantly suppress TAGLN expression both in the mRNA and protein levels [supplementary Fig. 9C-D]. Thus, we proposed that Src might indirectly regulate TAGLN expression by harnessing YAP.

Next, we investigated whether TAGLN could regulate Src activation. Silencing TAGLN markedly reduced Src activation [Fig. 5F], whereas overexpression of TAGLN noticeably enhanced the activation of Src in OC cells [Fig. 5G]. Consistently, we noted a significant reduction of phosphorylation of Src in TAGLN-silenced xenograft tumors [Fig. 5H], signifying that TAGLN could regulate Src activation *in vitro* and *in vivo*. This regulation loop was interesting, but how could TAGLN regulate Src gene activation, which was an mechanosensing molecular and supposed to be an up-streamer of TAGLN? During our experiment, we noticed that cells silencing TAGLN expression showed slimmer morphology [Fig. 5I]. and decreased cytoskeleton expression [Fig. 5J], indicating that TAGLN could modulate cellular mechanical environment, thereby regulating the mechanosensing molecule Src.

To demonstrate the correlation between Src and TAGLN in OC tissues, we analyzed phosphorylate-Src (Try416) and TAGLN expression in 10 primary tumors of HGSOC by western blot analysis. We also analyzed phosphorylate-Src (Try416) and TAGLN expression in 33 primary tumors of HGSOC patients by IHC staining. Pearson’s correlation confirmed the positive relationship between phosphorylate-Src (Try416) and TAGLN protein expression (*r* = 0.7403) [Fig. 5K]. Besides, we observed significantly lower phosphorylate-Src (Try416) expression in TAGLN-low group and higher expression in TAGLN-high group [Fig. 5L].

To sum up, our results demonstrated that mechanosensitive Src activation and TAGLN expression formed a regulation loop.

### TAGLN regulates ovarian cancer progression through RhoA/ROCK pathway

**Fig. 6:**
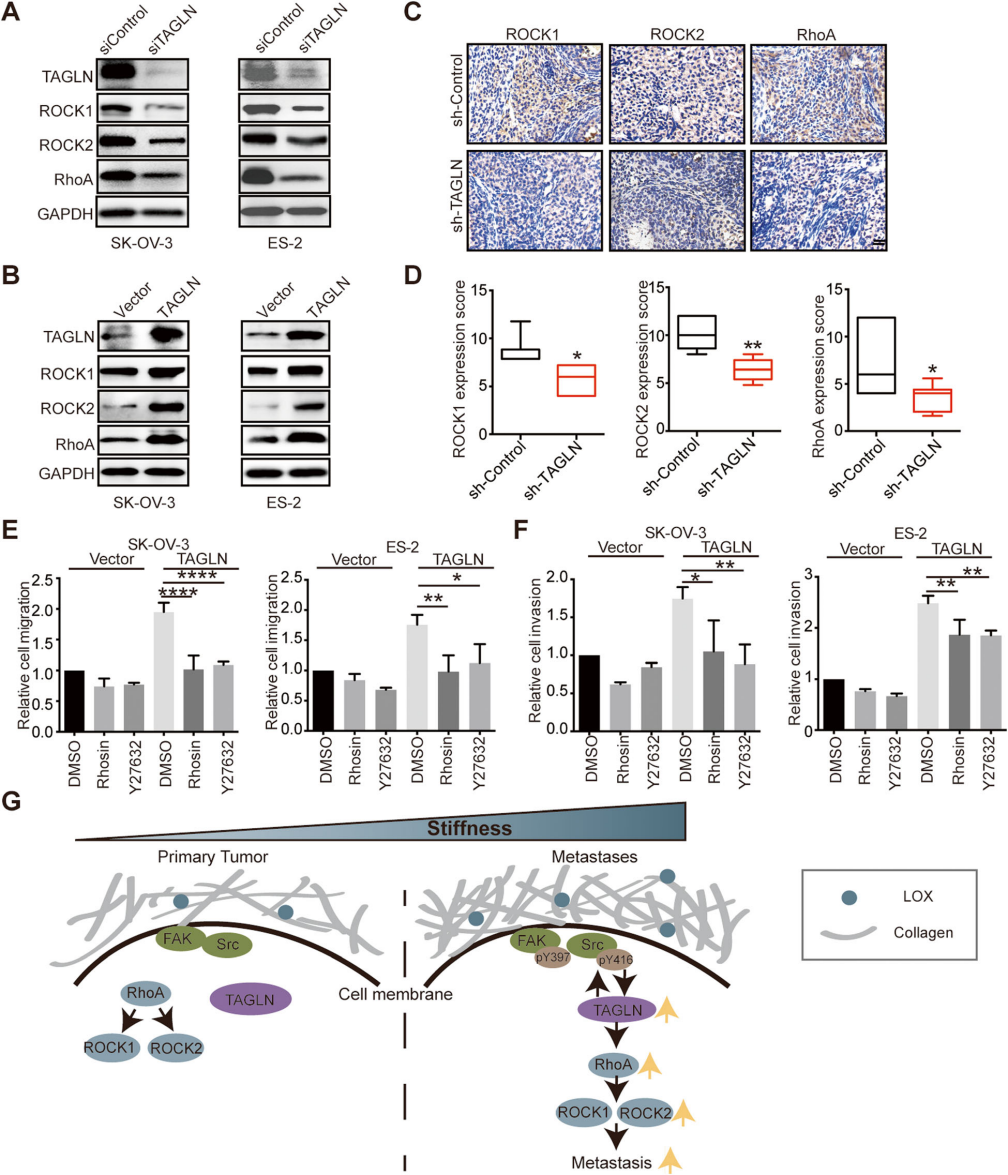
TAGLN regulates ovarian cancer progression through RhoA/ROCK pathway. **A** Western blot analyzed TAGLN, ROCK1, ROCK2 and RhoA of cells transfected with siControl or TAGLN siRNA. GAPDH was used as a loading control. **B** Western blot analyzed TAGLN, ROCK1, ROCK2 and RhoA of cells transfected with vector or TAGLN plasmid. GAPDH was used as a loading control. **C-D** Representative images (**C**) and scores (**D**)of IHC staining for ROCK1, ROCK2 and RhoA from mice bearing SK-OV-3 cells transfected with sh-control or sh-TAGLN lentivirus (*P < 0.05). Scale bar: 20 μm. **E-F** Migratory (**E)** and invasive (**F)** ability of cells treated with Rhosin or Y27632 after transfected with control vector or TAGLN plasmid (*P < 0.05, **P < 0.01, ****P < 0.0001). **G** A graphical illustration of the molecular signaling events involved in stiffness-regulated OC progression. TAGLN mediated stiffness-regulated OC progression through RhoA/ROCK pathway

Since RhoA/ROCK pathway was pivotal in cell motility and was activated upon mechanical cues in OC, we explored whether it was regulated by TAGLN to abet OC progression. Knockdown of TAGLN restrained RhoA/ROCK expression [Fig. 6A]. Moreover, overexpression of TAGLN remarkably increased RhoA/ROCK expression [Fig. 6B]. To verify it *in vivo*, we analyzed ROCK1, ROCK2 and RhoA expression in TAGLN-knockdown tumors, and observed that expressions of these proteins were decreased in sh-TAGLN group in contrast to sh-control group [Fig. 6 C-D]. Moreover, we found that both RhoA inhibitor (Rhosin) and ROCK inhibitor (Y27632) could inhibit the migration and invasion of OC cells, particularly for cells overexpressing TAGLN [Fig. 6E-F]. Taken together, these data demonstrated that TAGLN could regulate OC progression through RhoA/ROCK pathway.

## Discussion

In this study, we confirmed that omental metastases harbored higher stiffness and level of desmoplasia compared with primary tumors of EOC and demonstrated that matrix stiffness could modulate OC progression. Notably, we identified that TAGLN was a novel mechanosensitive gene that formed a regulation loop with Src, a pivotal mechanosignaling molecule. More importantly, TAGLN was revealed to mediate stiffness-regulated OC progression through RhoA/ROCK signaling pathway. Our study provided the evidence that targeting ECM stiffness could hamper OC progression, suggesting that TAGLN could be a promising therapeutic target for counteracting OC.

Increased matrix stiffness during tumor progression has been shown to contribute to malignant phenotypes of multiple cancers [7, 13, 21]. Here, by revealing that omental metastases of EOC were higher in stiffness than primary tumors, we uncovered the OC-promoting role of higher matrix stiffness and indicated the correlation between tissue mechanics and OC behaviors. We demonstrated that stiffer ECM could abet OC progression, suggesting that preventing or reversing tumor stiffening is a potential therapeutic strategy [8]. Targeting ECM stiffness using BAPN in our experiments significantly hampered OC progression, however, the high toxicity of BAPN in clinical trials precludes the clinical applications [28]. Alternative approaches such as inhibiting the LOX transcription factor have been proposed [29]. We revealed that the metastases of OC underwent more desmoplasia, resulting in higher stiffness, but the underlying mechanisms remain unclear. In the future, the investigations of the increased desmoplasia in metastases could provide other druggable targets for eliminating ECM stiffness.

Besides preventing ECM stiffening, an alternative method to overcome the adverse effects of microenvironment stiffening is to curb the cellular response to increased matrix mechanics that contribute to tumor malignant phenotypes [30]. Several researches have suggested the therapeutic effects of small molecule inhibitors that target mechanical signals in tumors, such as FAK inhibitor and YAP inhibitor [31, 32]. For example, small-molecule inhibitors that directly inhibit YAP/TAZ has been used as mechano-based interventions [31] and showed therapeutic effect in restraining ovarian cancer progression [33, 34]. Our previous study showed that targeting YAP using peptide 17 attenuated OC progression through down-regulation of PI3K/Akt/mTOR pathway [33]. Verteporfin, a suppressor of YAP–TEAD complex, has been proved to effectively reduce proliferation and inhibit the growth of OC cells in vitro and in vivo [34]. In our research, we also demonstrated that YAP could mediate Src activation regulated TAGLN expression, further revealed its role in ovarian cancer.

Notably, our study identified a novel mechanosensitive gene, TAGLN, which was demonstrated to form a regulation loop with Src activation. Src is a well-acknowledged mechanosignaling molecule that becomes activated upon physical signal [27]. Our study demonstrated that Src activation regulated TAGLN expression, therefore, we think Src was an up-streamer molecule of TAGLN. We discovered that TAGLN could in turn govern Src stimulation because silencing TAGLN reshaped the cellular morphology and cytoskeleton expression, could resulting in cellular tension alternation, therefore showed influence on Src activation.

Importantly, we revealed that TAGLN could mediate stiffness-regulated progression of ovarian malignancy through the regulation of RhoA/ROCK pathway and blocking TAGLN could diminish OC progression in the experiments. However, there is no approved or explored drug that targets TAGLN currently. Therefore, TAGLN holds great promise as a therapeutic target of OC.

## Conclusions

Our study highlighted the pivotal role of tissue mechanics in OC progression and supported that targeting ECM stiffness emerged as a promising strategy for OC treatment. Specifically, we revealed that TAGLN formed a regulation loop with Src activation and mediated stiffness-regulated OC progression through the regulation of RhoA/ROCK pathway, proposing that TAGLN was promising as a novel therapeutic target for suppressing OC progression.

## Supplementary Information

**Additional file 1: Supplementary Figure 1. **Matrix stiffness modulates ovarian cancer progression. (A) Phase images showing typical morphology of ES-2 cells cultured on soft and stiff collagen I coated PA gels. Scale bar: 50 μm. (B) Cell surface areas calculated by digital image analysis of phase-contrast images of ES-2 cells on soft and stiff collagen I coated PA gels (****P < 0.0001). (C) Representative immunofluorescence images (left) and quantifications (right) for phalloidin (red) and with DAPI (blue) (**P < 0.01). Scale bar: 20 μm. (D) Migratory (left) and invasive (right) ability of ES-2 cells cultured on soft versus stiff substrates evaluated by transwell assays (*P < 0.05). (E) The proliferation of ES-2 cells cultured on soft versus stiff substrates was measured by EdU assay, DAPI (blue). Scale bar: 50 μm. (right) Quantification of EdU-positive nuclei from *n* = 3 experiments (*P < 0.05)

**Additional file 2: Supplementary Figure 2.** Representative images (left) and scores of IHC staining (right) for Ki-67 from mice treated with saline or BAPN (*n* = 8 each group). Scale bar: 50 μm

**Additional file 3: Supplementary Figure 3. **Matrix stiffness activates Src gene and RhoA/ROCK pathway. (**A**) Western blot from cell lysates of ES-2 cells showing expression of p-Src (Try416), Src and GAPDH. GAPDH was used as a loading control. (**B**) Representative images (left) and scores (right) of IHC staining for p-Src (Try416) from mice treated with saline or BAPN (*n* = 8 each group, ****P < 0.0001). Scale bar: 20 μm. (**C**) Western blot from cell lysates of ES-2 cells on soft and stiff substrates showing expression of RhoA, ROCK1, ROCK2 and GAPDH. GAPDH was used as a loading control. Activated-RhoA was detected by RhoA pull down analysis. (**D-E**) Representative images of IHC staining (**D**) and quantification (**E**) for ROCK1, ROCK2 and RhoA from mice treated with saline or BAPN (*n* = 8 each group, *P < 0.05). Scale bar: 20 μm. Scale bar: 20 μm

**Additional file 4: Supplementary Figure 4.** “CYTOSKELETON” and “ACTIN_FILAMENT_BINDING” GSEA plot of enrichment of gene expression in our transcriptomic profiling

**Additional file 5: Supplementary Figure 5. **TAGLN expression correlated with substrates stiffness. (A) Transcriptomic profiling of SK-OV-3 cells cultured on soft (0.25 kPa) or stiff (40 kPa) PA gels. The heatmap shows genes upregulated in SK-OV-3 cells cultured on stiff (40 kPa) PA gels compared to soft (0.25 kPa) PA gels. Green or red in the heat map indicate genes expression that was relatively low or high, respectively. (B-C) Relative TAGLN (B), THBS1, OXTR (C) mRNA expression levels of SK-OV-3 and ES-2 cells cultured on substrates of different stiffness determined by quantitative PCR

**Additional file 6: Supplementary Figure 6.** Boxplots showing the expression level of TAGLN in paired ovarian dataset GSE30587 and unpaired ovarian dataset GSE2109

**Additional file 7: Supplementary Figure 7. **TAGLN mediates stiffness-regulated ovarian cancer progression. (A) Knockdown of TAGLN by siRNA in ES-2 cells were determined by western blot and probed for TAGLN and GAPDH. (B-C) Migratory (B) and invasive (C) ability of ES-2 cells transfected with siControl or TAGLN siRNA, cultured on soft or stiff substrates, were evaluated by transwell assays and quantified (*P < 0.05, **P < 0.01). (D) Overexpression of TAGLN by plasmid transfection in ES-2 cells were determined by western blot and probed for TAGLN and GAPDH. (E-F) Migratory (E) and invasive (F) ability of ES-2 cells with the expression of control vector or overexpression of TAGLN protein, cultured on soft or stiff substrates, were evaluated by transwell assays and quantified (*P < 0.05, **P < 0.01)

**Additional file 8: Supplementary Figure 8. **TAGLN correlates with poor patient prognosis. (A) Survival z-scores in different cancer types associated with expression of TAGLN mRNA. Red indicates poor survival and blue indicates good survival. (B-C) Meta-analysis depicting the forest plots of TAGLN expression as a univariate predictor of PFS (B) and OS (C), using several datasets with applicable genes expression and survival information of OC patients

**Additional file 9: Supplementary Figure 9. **Src activation might regulate TAGLN via YAP. (A) Western blot analyzed YAP and p-Src (Try416) expression of cells treated with dasatinib. GAPDH was used as a loading control. (B) Western blot analyzed YAP and p-Src (Try416) expression of cells treated with Src inhibitor 1. GAPDH was used as a loading control. (C) Relative TAGLN mRNA expression levels of SK-OV-3 and ES-2 cells transfected with siControl or siYAP. (D)Western blot analyzed TAGLN and YAP expression of cells transfected with siControl or siYAP. GAPDH was used as a loading control

**Additional file 10:** Supplementary Methods

**Additional file 11: Supplementary Table 1. **Details on the antibodies used in the present study. **Supplementary Table 2. **Details on the reagents used in the present study. **Supplementary Table 3.** Individual Genes Used for Analysis of GSE30587 and GSE2109. **Supplementary Table 4.** Patient characteristics used in Fig. 1. **Supplementary Table 5.** Patient characteristics related to Fig. 3. **Supplementary Table 6.** Patient characteristics related to Fig. 4. **Supplementary Table 7.** Patient characteristics related to Fig. 5. **Supplementary Table 8.** Patient characteristics related to supplementary Fig. 2. 

## Data Availability

Previously published microarray data that were reanalyzed here are available from the TCGA Research Network (http://cancergenome.nih.gov) via download from the CBio Portal for Cancer Genomics (http://www.cbioportal.org/public-portal/index.do) under the Ovarian Serous Cystadenocarcinoma data sets. For correlation analysis, statistical significance was calculated by Pearson’s correlation analysis. To explore the differentially expression of genes in primary and metastatic ovarian cancer tissues, we utilized gene expression data (GSE30587, GSE2109 profiling data), which were downloaded as series matrix data from Gene Expression Omnibus (http://www.ncbi.nlm.nih.gov/geo). The Kaplan-Meier plotter tool (http://kmplot.com/analysis/) was used to generate survival curves combining TAGLN (Affymetrix probe 205547_s_at) mRNA data from all public ovarian cancer datasets. Analysis of TAGLN expression and cancer outcomes was using PREdiction of Clinical Outcomes from Genomic Profiles (PRECOG) (https://precog.stanford.edu/).

## Abbreviations

BAPN: β-aminopropionitrile
ECM: Extracellular matrix
IHC: Immunohistochemistry
LOX: Lysyl oxidase
OS: Overall survival
PFS: Progress free survival
OC: ovarian cancer
TAGLN: Transgelin
ROCK1: Rho associated coiled-coil containing protein kinase1
ROCK2: Rho associated coiled-coil containing protein kinase 2
AFM: Atomic force microscope
YAP: Yes-associated protein

## References

[1] Lengyel E (2010). Ovarian cancer development and metastasis. Am J Pathol.

[2] Nieman KM, Kenny HA, Penicka CV, Ladanyi A, Buell-Gutbrod R, Zillhardt MR, Romero IL, Carey MS, Mills GB, Hotamisligil GS (2011). Adipocytes promote ovarian cancer metastasis and provide energy for rapid tumor growth. Nat Med.

[3] Yin M, Li X, Tan S, Zhou HJ, Ji W, Bellone S, Xu X, Zhang H, Santin AD, Lou G (2016). Tumor-associated macrophages drive spheroid formation during early transcoelomic metastasis of ovarian cancer. J Clin Investig.

[4] Gao Q, Yang Z, Xu S, Li X, Yang X, Jin P, Liu Y, Zhou X, Zhang T, Gong C (2019). Heterotypic CAF-tumor spheroids promote early peritoneal metastatis of ovarian cancer. J Exp Med.

[5] Paszek MJ, Zahir N, Johnson KR, Lakins JN, Rozenberg GI, Gefen A, Reinhart-King CA, Margulies SS, Dembo M, Boettiger D (2005). Tensional homeostasis and the malignant phenotype. Cancer Cell.

[6] Acerbi I, Cassereau L, Dean I, Shi Q, Au A, Park C, Chen YY, Liphardt J, Hwang ES, Weaver VM (2015). Human breast cancer invasion and aggression correlates with ECM stiffening and immune cell infiltration. Integr Biol (Camb).

[7] Miroshnikova YA, Mouw JK, Barnes JM, Pickup MW, Lakins JN, Kim Y, Lobo K, Persson AI, Reis GF, McKnight TR (2016). Tissue mechanics promote IDH1-dependent HIF1alpha-tenascin C feedback to regulate glioblastoma aggression. Nat Cell Biol.

[8] Mohammadi H, Sahai E (2018). Mechanisms and impact of altered tumour mechanics. Nat Cell Biol.

[9] Levental KR, Yu H, Kass L, Lakins JN, Egeblad M, Erler JT, Fong SF, Csiszar K, Giaccia A, Weninger W (2009). Matrix crosslinking forces tumor progression by enhancing integrin signaling. Cell.

[10] Wei SC, Fattet L, Tsai JH, Guo Y, Pai VH, Majeski HE, Chen AC, Sah RL, Taylor SS, Engler AJ (2015). Matrix stiffness drives epithelial-mesenchymal transition and tumour metastasis through a TWIST1-G3BP2 mechanotransduction pathway. Nat Cell Biol.

[11] Barnes JM, Kaushik S, Bainer RO, Sa JK, Woods EC, Kai F, Przybyla L, Lee M, Lee HW, Tung JC (2018). A tension-mediated glycocalyx-integrin feedback loop promotes mesenchymal-like glioblastoma. Nat Cell Biol.

[12] Schrader J, Gordon-Walker TT, Aucott RL, van Deemter M, Quaas A, Walsh S, Benten D, Forbes SJ, Wells RG, Iredale JP (2011). Matrix stiffness modulates proliferation, chemotherapeutic response, and dormancy in hepatocellular carcinoma cells. Hepatology.

[13] Mouw JK, Yui Y, Damiano L, Bainer RO, Lakins JN, Acerbi I, Ou G, Wijekoon AC, Levental KR, Gilbert PM (2014). Tissue mechanics modulate microRNA-dependent PTEN expression to regulate malignant progression. Nat Med.

[14] Provenzano PP, Cuevas C, Chang AE, Goel VK, Von Hoff DD, Hingorani SR (2012). Enzymatic targeting of the stroma ablates physical barriers to treatment of pancreatic ductal adenocarcinoma. Cancer Cell.

[15] Dupont S, Morsut L, Aragona M, Enzo E, Giulitti S, Cordenonsi M, Zanconato F, Le Digabel J, Forcato M, Bicciato S (2011). Role of YAP/TAZ in mechanotransduction. Nature.

[16] Wei SC, Yang J (2016). Forcing through tumor metastasis: the interplay between tissue rigidity and epithelial-mesenchymal transition. Trends Cell Biol.

[17] Cancer Genome Atlas Research N (2011). Integrated genomic analyses of ovarian carcinoma. Nature.

[18] Wei X, Wei R, Jiang G, Jia Y, Lou H, Yang Z, Luo D, Huang Q, Xu S, Yang X (2019). Mechanical cues modulate cellular uptake of nanoparticles in cancer via clathrin-mediated and caveolae-mediated endocytosis pathways. Nanomedicine.

[19] Plodinec M, Lim RY (2015). Nanomechanical characterization of living mammary tissues by atomic force microscopy. Methods Mol Biol.

[20] Zhao X, Fang Y, Yang Y, Qin Y, Wu P, Wang T, Lai H, Meng L, Wang D, Zheng Z (2015). Elaiophylin, a novel autophagy inhibitor, exerts antitumor activity as a single agent in ovarian cancer cells. Autophagy.

[21] Laklai H, Miroshnikova YA, Pickup MW, Collisson EA, Kim GE, Barrett AS, Hill RC, Lakins JN, Schlaepfer DD, Mouw JK (2016). Genotype tunes pancreatic ductal adenocarcinoma tissue tension to induce matricellular fibrosis and tumor progression. Nat Med.

[22] Tse JR, Engler AJ. Preparation of hydrogel substrates with tunable mechanical properties. Curr Prot Cell Biol. 2010, Chap. 10:Unit 10.16. 10.1002/0471143030.cb1016s47 20521229

[23] Butcher DT, Alliston T, Weaver VM (2009). A tense situation: forcing tumour progression. Nat Rev Cancer.

[24] Boyle ST, Samuel MS (2016). Mechano-reciprocity is maintained between physiological boundaries by tuning signal flux through the Rho-associated protein kinase. Small GTPases.

[25] Gentles AJ, Newman AM, Liu CL, Bratman SV, Feng W, Kim D, Nair VS, Xu Y, Khuong A, Hoang CD (2015). The prognostic landscape of genes and infiltrating immune cells across human cancers. Nat Med.

[26] Ganzfried BF, Riester M, Haibe-Kains B, Risch T, Tyekucheva S, Jazic I, Wang XV, Ahmadifar M, Birrer MJ, Parmigiani G, et al. curatedOvarianData: clinically annotated data for the ovarian cancer transcriptome. Database. 2013;2013:bat013. 10.1093/database/bat013 PMC3625954 23550061

[27] Na S, Collin O, Chowdhury F, Tay B, Ouyang M, Wang Y, Wang N (2008). Rapid signal transduction in living cells is a unique feature of mechanotransduction. Proc Natl Acad Sci U S A.

[28] Keiser HR, Sjoerdsma A (1967). Studies on beta-aminopropionitrile in patients with scleroderma. Clin Pharmacol Ther.

[29] Cox TR, Gartland A, Erler JT (2016). Lysyl oxidase, a targetable secreted molecule involved in cancer metastasis. Cancer Res.

[30] Lampi MC, Reinhart-King CA. Targeting extracellular matrix stiffness to attenuate disease: From molecular mechanisms to clinical trials. Sci Transl Med. 2018;10(422):eaao0475. 10.1126/scitranslmed.aao0475 29298864

[31] Liu-Chittenden Y, Huang B, Shim JS, Chen Q, Lee SJ, Anders RA, Liu JO, Pan D (2012). Genetic and pharmacological disruption of the TEAD-YAP complex suppresses the oncogenic activity of YAP. Genes Dev.

[32] Golubovskaya VM, Figel S, Ho BT, Johnson CP, Yemma M, Huang G, Zheng M, Nyberg C, Magis A, Ostrov DA (2012). A small molecule focal adhesion kinase (FAK) inhibitor, targeting Y397 site: 1-(2-hydroxyethyl)-3, 5, 7-triaza-1-azoniatricyclo [3.3.1.1(3,7)]decane; bromide effectively inhibits FAK autophosphorylation activity and decreases cancer cell viability, clonogenicity and tumor growth in vivo. Carcinogenesis.

[33] Wei X, Jia Y, Lou H, Ma J, Huang Q, Meng Y, Sun C, Yang Z, Li X, Xu S (2019). Targeting YAP suppresses ovarian cancer progression through regulation of the PI3K/Akt/mTOR pathway. Oncol Rep.

[34] Feng J, Gou J, Jia J, Yi T, Cui T, Li Z (2016). Verteporfin, a suppressor of YAP-TEAD complex, presents promising antitumor properties on ovarian cancer. Onco Targets Ther.

