# Supplementary figures and images for: TAGLN mediated stiffness-regulated ovarian cancer progression via RhoA/ROCK pathway
Source: J Exp Clin Cancer Res. 2021 Sep 19;40:292. doi: 10.1186/s13046-021-02091-6 (PMC8451140; doi:10.1186/s13046-021-02091-6)

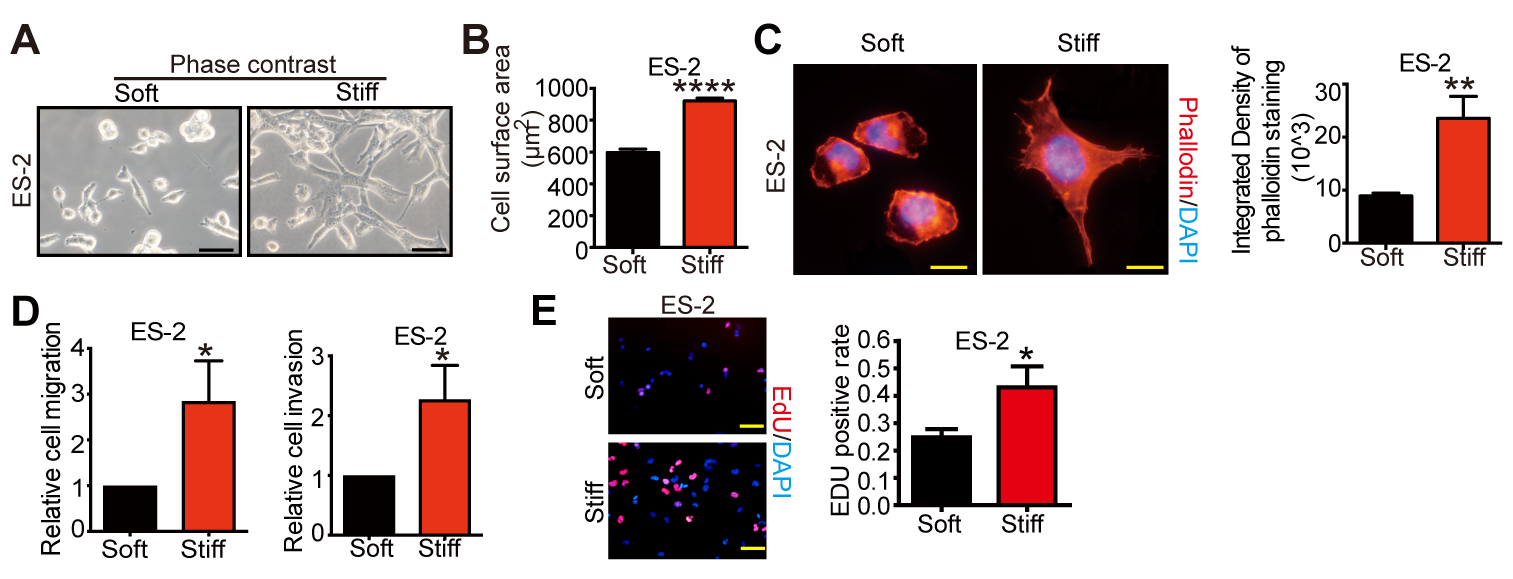

Supplement: Supplementary file 1 — Additional file 1: Supplementary Figure 1. Matrix stiffness modulates ovarian cancer progression. (A) Phase images showing typical morphology of ES-2 cells cultured on soft and stiff collagen I coated PA gels. Scale bar: 50 μm. (B) Cell surface areas calculated by digital image analysis of phase-contrast images of ES-2 cells on soft and stiff collagen I coated PA gels (****P < 0.0001). (C) Representative immunofluorescence images (left) and quantifications (right) for phalloidin (red) and with DAPI (blue) (**P < 0.01). Scale bar: 20 μm. (D) Migratory (left) and invasive (right) ability of ES-2 cells cultured on soft versus stiff substrates evaluated by transwell assays (*P < 0.05). (E) The proliferation of ES-2 cells cultured on soft versus stiff substrates was measured by EdU assay, DAPI (blue). Scale bar: 50 μm. (right) Quantification of EdU-positive nuclei from n = 3 experiments (*P < 0.05) [file 13046_2021_2091_MOESM1_ESM.tif]

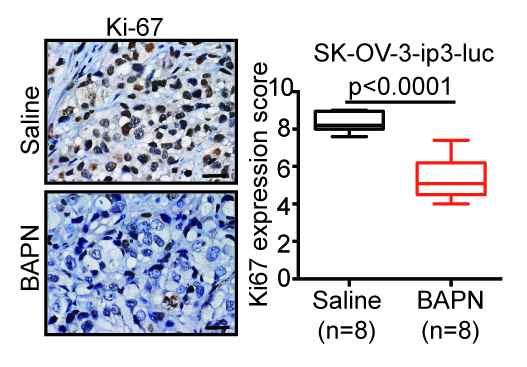

Supplement: Supplementary file 2 — Additional file 2: Supplementary Figure 2. Representative images (left) and scores of IHC staining (right) for Ki-67 from mice treated with saline or BAPN (n = 8 each group). Scale bar: 50 μm [file 13046_2021_2091_MOESM2_ESM.tif]

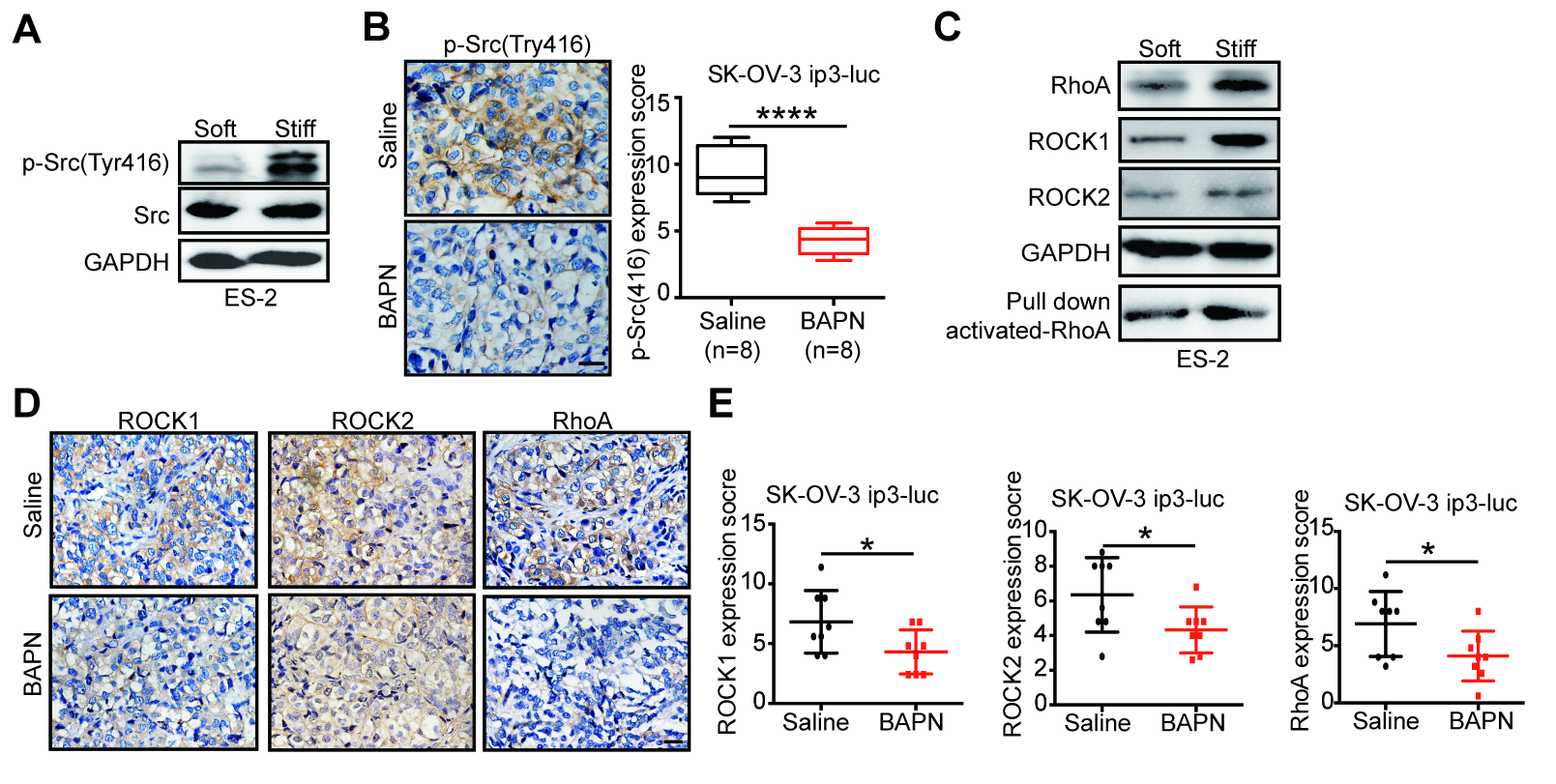

Supplement: Supplementary file 3 — Additional file 3: Supplementary Figure 3. Matrix stiffness activates Src gene and RhoA/ROCK pathway. (A) Western blot from cell lysates of ES-2 cells showing expression of p-Src (Try416), Src and GAPDH. GAPDH was used as a loading control. (B) Representative images (left) and scores (right) of IHC staining for p-Src (Try416) from mice treated with saline or BAPN (n = 8 each group, ****P < 0.0001). Scale bar: 20 μm. (C) Western blot from cell lysates of ES-2 cells on soft and stiff substrates showing expression of RhoA, ROCK1, ROCK2 and GAPDH. GAPDH was used as a loading control. Activated-RhoA was detected by RhoA pull down analysis. (D-E) Representative images of IHC staining (D) and quantification (E) for ROCK1, ROCK2 and RhoA from mice treated with saline or BAPN (n = 8 each group, *P < 0.05). Scale bar: 20 μm. Scale bar: 20 μm [file 13046_2021_2091_MOESM3_ESM.tif]

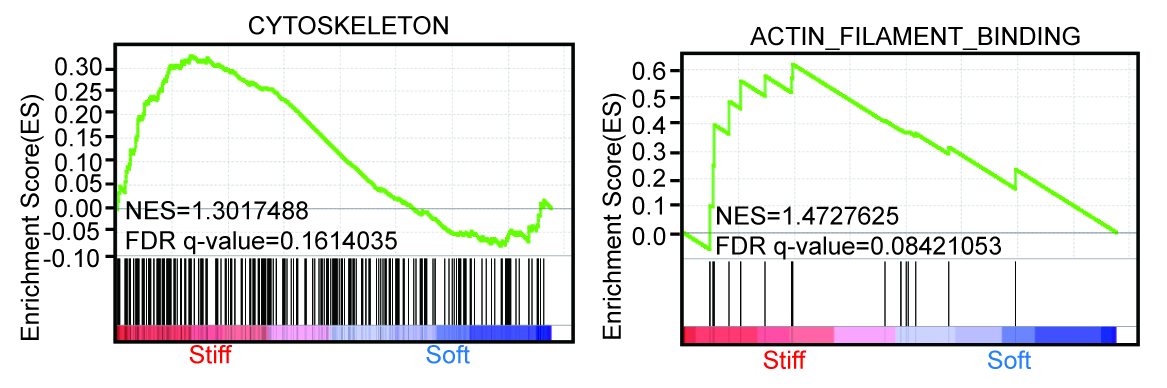

Supplement: Supplementary file 4 — Additional file 4: Supplementary Figure 4. “CYTOSKELETON” and “ACTIN_FILAMENT_BINDING” GSEA plot of enrichment of gene expression in our transcriptomic profiling [file 13046_2021_2091_MOESM4_ESM.tif]

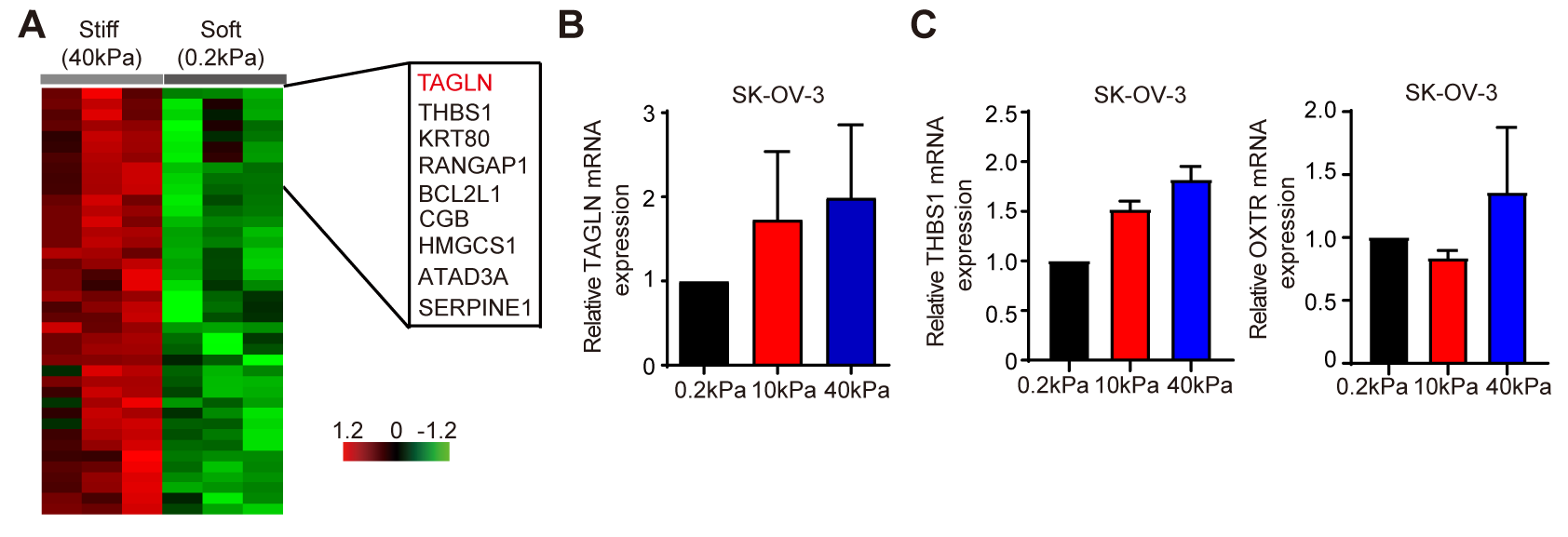

Supplement: Supplementary file 5 — Additional file 5: Supplementary Figure 5. TAGLN expression correlated with substrates stiffness. (A) Transcriptomic profiling of SK-OV-3 cells cultured on soft (0.25 kPa) or stiff (40 kPa) PA gels. The heatmap shows genes upregulated in SK-OV-3 cells cultured on stiff (40 kPa) PA gels compared to soft (0.25 kPa) PA gels. Green or red in the heat map indicate genes expression that was relatively low or high, respectively. (B-C) Relative TAGLN (B), THBS1, OXTR (C) mRNA expression levels of SK-OV-3 and ES-2 cells cultured on substrates of different stiffness determined by quantitative PCR [file 13046_2021_2091_MOESM5_ESM.tif]

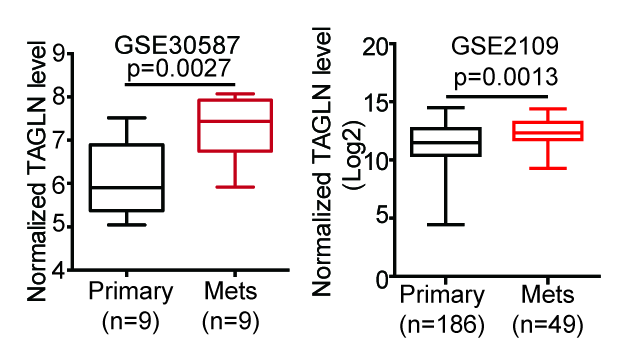

Supplement: Supplementary file 6 — Additional file 6: Supplementary Figure 6. Boxplots showing the expression level of TAGLN in paired ovarian dataset GSE30587 and unpaired ovarian dataset GSE2109 [file 13046_2021_2091_MOESM6_ESM.tif]

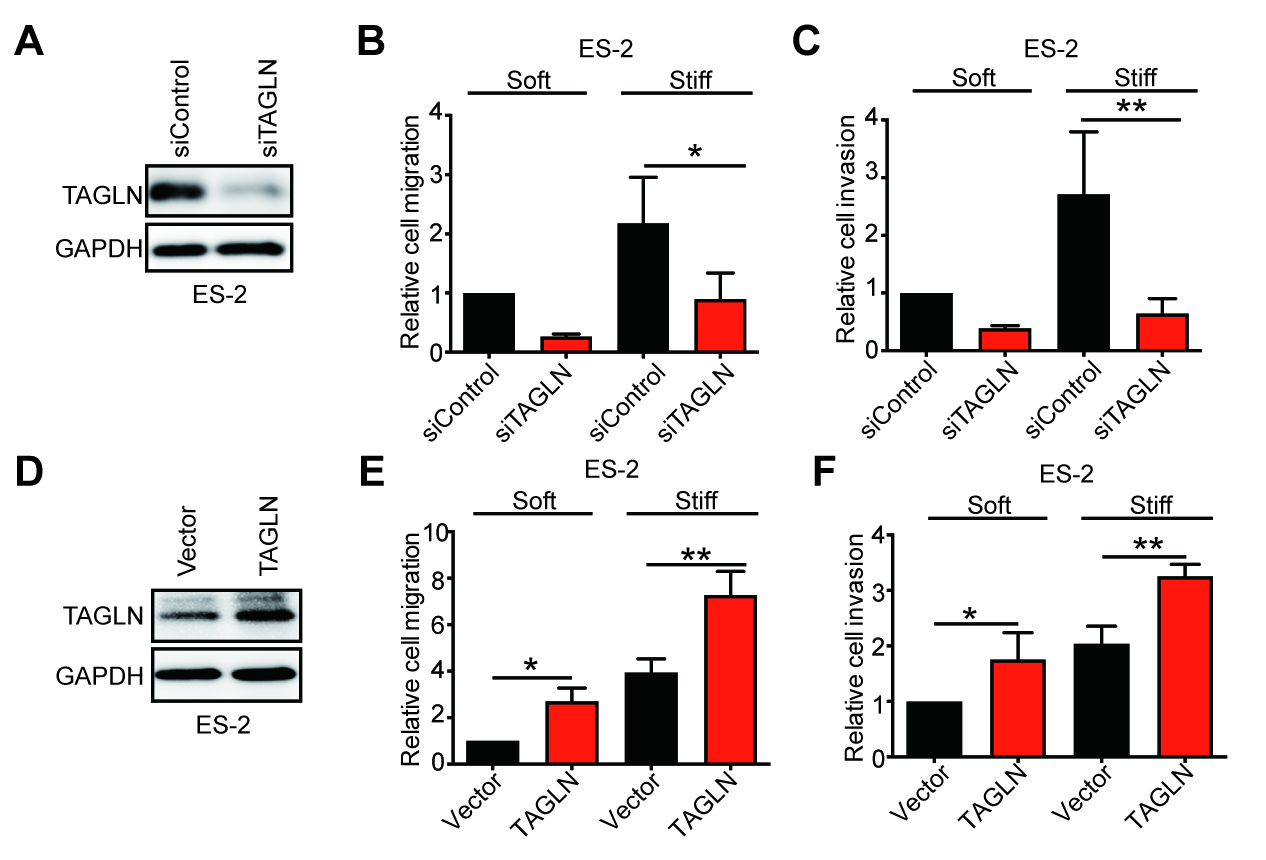

Supplement: Supplementary file 7 — Additional file 7: Supplementary Figure 7. TAGLN mediates stiffness-regulated ovarian cancer progression. (A) Knockdown of TAGLN by siRNA in ES-2 cells were determined by western blot and probed for TAGLN and GAPDH. (B-C) Migratory (B) and invasive (C) ability of ES-2 cells transfected with siControl or TAGLN siRNA, cultured on soft or stiff substrates, were evaluated by transwell assays and quantified (*P < 0.05, **P < 0.01). (D) Overexpression of TAGLN by plasmid transfection in ES-2 cells were determined by western blot and probed for TAGLN and GAPDH. (E-F) Migratory (E) and invasive (F) ability of ES-2 cells with the expression of control vector or overexpression of TAGLN protein, cultured on soft or stiff substrates, were evaluated by transwell assays and quantified (*P < 0.05, **P < 0.01) [file 13046_2021_2091_MOESM7_ESM.tif]

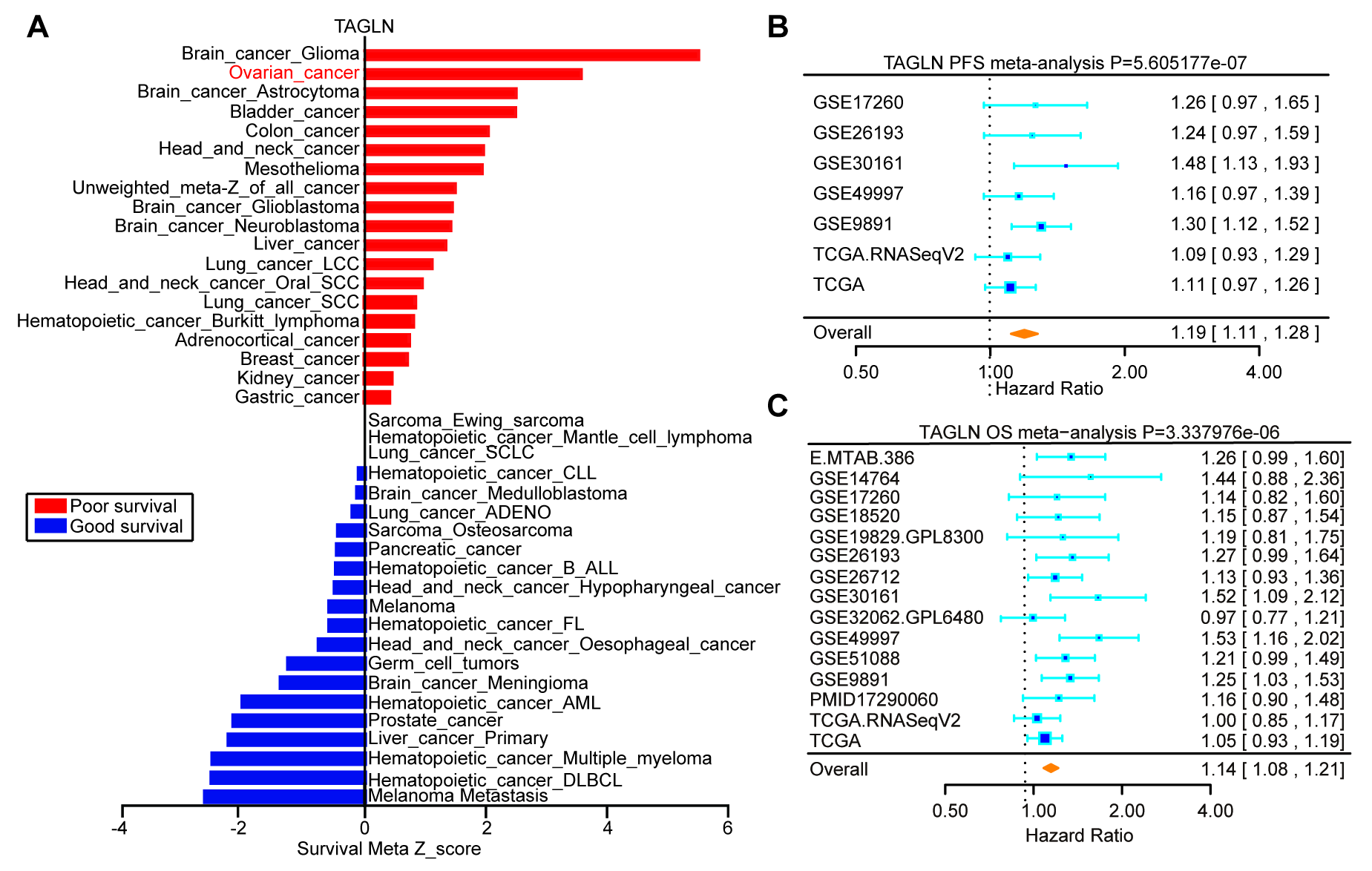

Supplement: Supplementary file 8 — Additional file 8: Supplementary Figure 8. TAGLN correlates with poor patient prognosis. (A) Survival z-scores in different cancer types associated with expression of TAGLN mRNA. Red indicates poor survival and blue indicates good survival. (B-C) Meta-analysis depicting the forest plots of TAGLN expression as a univariate predictor of PFS (B) and OS (C), using several datasets with applicable genes expression and survival information of OC patients [file 13046_2021_2091_MOESM8_ESM.tif]

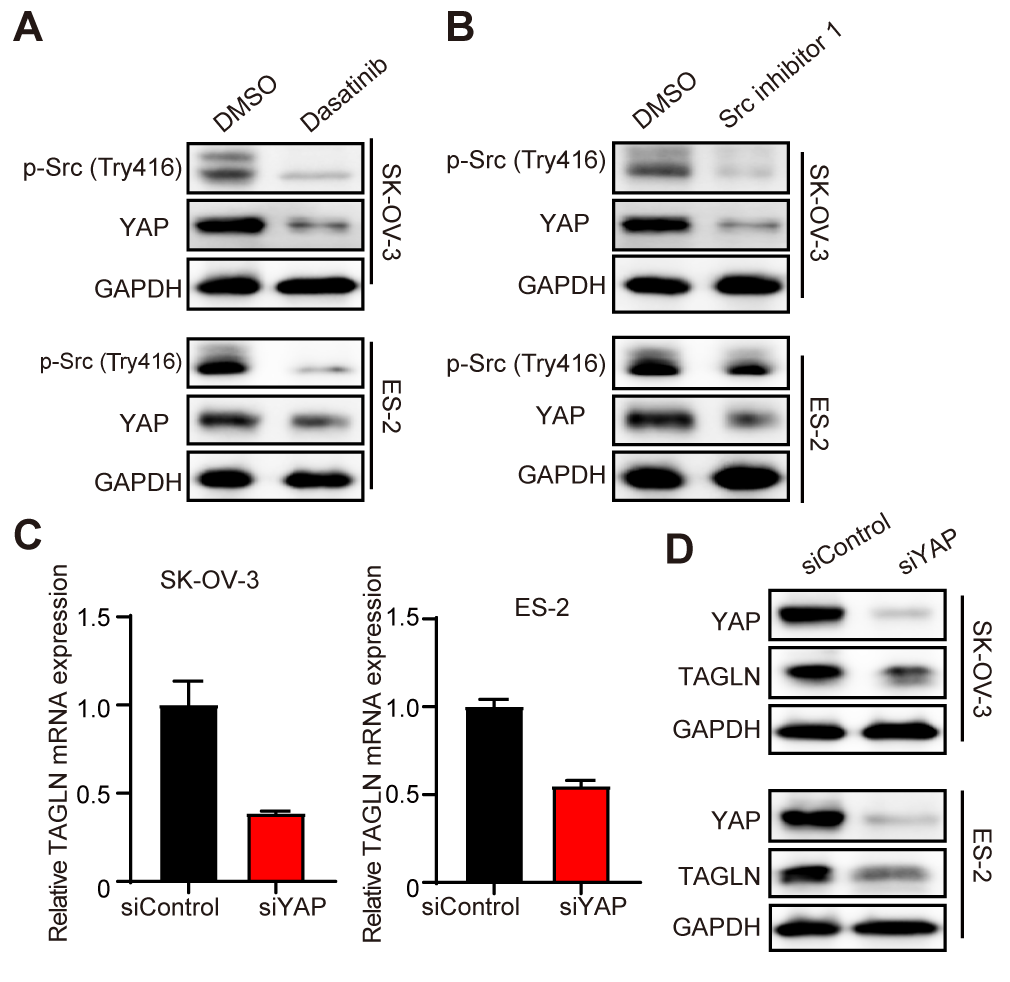

Supplement: Supplementary file 9 — Additional file 9: Supplementary Figure 9. Src activation might regulate TAGLN via YAP. (A) Western blot analyzed YAP and p-Src (Try416) expression of cells treated with dasatinib. GAPDH was used as a loading control. (B) Western blot analyzed YAP and p-Src (Try416) expression of cells treated with Src inhibitor 1. GAPDH was used as a loading control. (C) Relative TAGLN mRNA expression levels of SK-OV-3 and ES-2 cells transfected with siControl or siYAP. (D)Western blot analyzed TAGLN and YAP expression of cells transfected with siControl or siYAP. GAPDH was used as a loading control [file 13046_2021_2091_MOESM9_ESM.tif]
