# Supplementary material for: TAGLN mediated stiffness-regulated ovarian cancer progression via RhoA/ROCK pathway
Source: J Exp Clin Cancer Res. 2021 Sep 19;40:292. doi: 10.1186/s13046-021-02091-6 (PMC8451140; doi:10.1186/s13046-021-02091-6)
Supplement: Supplementary file 10 — Additional file 10: Supplementary Methods [file 13046_2021_2091_MOESM10_ESM.docx]

**Supplementary Methods**

**Masson and picrosirius red staining**

Paraffin-embedded samples were sectioned at 5 μm and Masson’s trichrome staining was performed as described(1). Sections were imaged with an Olympus microscope. Five fields of each section were acquired. Further quantitative morphometric analysis was using Image-pro plus 6.0 (Media Cybernetics, Inc., Rockville, MD, USA). The paraffin-embedded sections of ovarian cancer were stained with 0.1% picrosirius red (Direct Red 80, Sigma) and counterstained with Weigert’s hematoxylin to reveal fibrillar collagen as described(2). Sections were serially imaged with an NIKON Eclipse Ci fluorescence microscope fitted with an analyzer (C-ISA, NIKON) and polarizer (C-SP, NIKON) oriented parallel and orthogonal to each other and quantified with minimal threshold. For analysis of collagen intensity, 5 images per tissues were taken for all patients and further quantified with Image-pro plus 6.0 (Media Cybernetics, Inc., Rockville, MD, USA). The results per tissue were averaged.

**Immunohistochemistry**

Standard immunohistochemistry techniques were performed as described previously(3). Paraffin-embedded tissue sections were first deparaffinized. After antigen retrieval, incubate slides with 3% H_2_O_2_ to inhibit endogenous peroxides, and then blocked with bovine serum for 30 min and incubated with primary antibody at 4℃ overnight. The dilution of primary antibodies was proved in supplementary table 1-2. Briefly, the staining intensity was graded (0, absence; 1, weak; 2, moderate; 3, strong). The percentage of positive tumor cells were scored as follows: graded as 1, no detectable immunostaining; 2, focal, <25% of cells immunostained; 3, regional, between 25% and 50% of cells immunostained; 4, diffuse, >50% of cells immunostained(4). Two independent observers that were blind to the details of the specimens evaluated the Immunostaining intensity. Statistical significance analysis was described in the statistical analysis.

**Quantitative real-time PCR**

Total RNA was isolated using the RNAsimple total RNA isolation kit (TIANGEN, DP419) according to the manufacturer’s instructions. Reverse transcription was performed from 2 μg of total RNA using random primers (Takara, Japan). The relative quantity of mRNA was determined by real-time RT-PCR as described previously(5). The primer sequences were as follows: for GAPDH: forward, 5’-ATG GAA ATC CCA TCA CCA TCT T-3’; reverse, 5’-CGC CCC ACT TGA TTT TGG -3’, TAGLN: forward: 5’-AGT GCA GTC CAA AAT CGA GAA G-3’; reverse: 5’-CTT GCT CAG AAT CAC GCC AT-3’. YAP forward, 5'-TAG CCC TGC GTA GCC AGT TA-3' and reverse, 5'-TCA TGC TTA GTC CAC TGT CTG T-3'. The comparative Cq method was used to calculate the relative mRNA expression levels. The expression level of GAPDH was used as a loading control.

**Western blot analysis**

Western blot analysis was performed as described previously(5). Antibodies used for immunoblotting analysis are listed in the Antibodies and reagents section. GAPDH was utilized as the internal control. Quantitation of protein expression was determined by image J.

**Transwell assay**

Migration and Matrigel invasion assays were performed as described previously(5). The migrated and invaded cells were photographed and quantified under an Olympus microscope.

**Rho GTPase activity**

The level of activated RhoA was determined using a Rhoteking-binding assay kit according to the manufacture (Cell Biolabs; STA-403-A). Lysates were centrifuged and then incubated with Rhotekin RBD agarose at 4℃ for 1 h. Bead was washed with assay buffer and resuspended with SDS-PAGE sample buffer. Analyze the eluate by western blot with a monoclonal anti-RhoA antibody.

**Gene set enrichment analysis**

To determine the enrichment of specific gene signatures in the genesets in the profiling of our transcriptomic microarray, gene set enrichment analysis (GSEA) was executed using the publicly available desktop application from the Broad Institute (http:// www.broad.mit.edu/gsea/software/software_index.html). Genesets such as “CYTOSKELETON” and “ACTIN-FILAMENT-BINDING” were selected from MSIGDB signature datasets.

**Transcriptomic array**

SK-OV-3 cells were cultured on PA gels of 0.5kPa or 10kPa for 24 h, and then trypsinized from PA gels. Total RNA was quantified by the Nano Drop ND-2000 (Thermo Scientific) and the RNA integrity was assessed using Agilent Bioanalyzer 2100(Agilent Technologies). The Human Genome U133 Plus 2.0 Array (Affymetrix) was utilized gene expression profiling. The sample labeling, microarray hybridization and washing were performed based on the manufacturer’s standard protocols. Briefly, total RNA was transcribed to double strand cDNA, then synthesized cRNA and labeled with biotin. The labeled cRNAs were hybridized onto the microarray. After washing and staining, the arrays were scanned by the Affymetrix Scanner 3000 (Affymetrix). Affymetrix Gene Chip Command Console (version 4.0, Affymetrix) was used to analyze array images to get raw data and the raw data was normalized with the RMA algorithm using Genespring software (version 13.1; Agilent Technologies). The threshold set for up- and down-regulated genes was a fold change>= 2.0 and a P value<= 0.05. Hierarchical Clustering was performed to display the distinguishable genes' expression pattern among samples.

**Data availability**

Previously published microarray data(6) that were reanalyzed here are available from the TCGA Research Network (http:// cancergenome.nih.gov) via download from the CBio Portal for Cancer Genomics (http://www.cbioportal.org/public-portal/index.do) under the Ovarian Serous Cystadenocarcinoma data sets. For correlation analysis, statistical significance was calculated by Pearson’s correlation analysis. To explore the differentially expression of genes in primary and metastatic ovarian cancer tissues, we utilized gene expression data (GSE30587, GSE2109 profiling data), which were downloaded as series matrix data from Gene Expression Omnibus (http:// www.ncbi.nlm.nih.gov/geo). The Kaplan-Meier plotter tool (http://kmplot.com/analysis/) was used to generate survival curves combining TAGLN (Affymetrix probe 205547_s_at) mRNA data from all public ovarian cancer datasets(7). Analysis of TAGLN expression and cancer outcomes was using PREdiction of Clinical Outcomes from Genomic Profiles (PRECOG) (<https://precog.stanford.edu/>).

**References:**

1. Acerbi I, Cassereau L, Dean I, Shi Q, Au A, Park C*, et al.* Human breast cancer invasion and aggression correlates with ECM stiffening and immune cell infiltration. Integrative biology : quantitative biosciences from nano to macro **2015**;7:1120-34

2. Levental KR, Yu H, Kass L, Lakins JN, Egeblad M, Erler JT*, et al.* Matrix crosslinking forces tumor progression by enhancing integrin signaling. Cell **2009**;139:891-906

3. Takai K, Le A, Weaver VM, Werb Z. Targeting the cancer-associated fibroblasts as a treatment in triple-negative breast cancer. Oncotarget **2016**;7:82889-901

4. Ji T, Gong D, Han Z, Wei X, Yan Y, Ye F*, et al.* Abrogation of constitutive Stat3 activity circumvents cisplatin resistant ovarian cancer. Cancer letters **2013**;341:231-9

5. Wei X, Liu Y, Gong C, Ji T, Zhou X, Zhang T*, et al.* Targeting Leptin as a Therapeutic Strategy against Ovarian Cancer Peritoneal Metastasis. Anti-cancer agents in medicinal chemistry **2016**

6. Cancer Genome Atlas Research N. Integrated genomic analyses of ovarian carcinoma. Nature **2011**;474:609-15

7. Gyorffy B, Lanczky A, Szallasi Z. Implementing an online tool for genome-wide validation of survival-associated biomarkers in ovarian-cancer using microarray data from 1287 patients. Endocrine-related cancer **2012**;19:197-208
