# Supplementary material for: TAGLN mediated stiffness-regulated ovarian cancer progression via RhoA/ROCK pathway
Source: J Exp Clin Cancer Res. 2021 Sep 19;40:292. doi: 10.1186/s13046-021-02091-6 (PMC8451140; doi:10.1186/s13046-021-02091-6)
Supplement: Supplementary file 11 — Additional file 11: Supplementary Table 1. Details on the antibodies used in the present study. Supplementary Table 2. Details on the reagents used in the present study. Supplementary Table 3. Individual Genes Used for Analysis of GSE30587 and GSE2109. Supplementary Table 4. Patient characteristics used in Fig. 1. Supplementary Table 5. Patient characteristics related to Fig. 3. Supplementary Table 6. Patient characteristics related to Fig. 4. Supplementary Table 7. Patient characteristics related to Fig. 5. Supplementary Table 8. Patient characteristics related to supplementary Fig. 2. [file 13046_2021_2091_MOESM11_ESM.doc]

**Supplementary Table 1. Details on the** antibodies used in the present study.

| **Antibodies** | **Source** | **Dilution** | **Catalogue number** | **Manufacturer** |
| --- | --- | --- | --- | --- |
| Anti-LOX antibody | Rabbit | WB: 1:1000;  IHC: 1:200 | ab174316 | Abcam |
| Anti-Collagen I antibody | Rabbit | WB:1:1000; IHC:1:200 | ab138492 | Abcam |
| Anti-ROCK1 antibody | Rabbit | WB: 1:1000  IHC:1:100 | ab45171 | Abcam |
| Anti-ROCK2 antibody | Rabbit | WB: 1:1000 | ab125025 | Abcam |
| Anti-RhoA antibody | Rabbit | WB: 1:4,000  IHC: 1:100 | ab187027 | Abcam |
| Anti-CDC42 antibody | Rabbit | WB: 1:1000 | ab187643 | Abcam |
| Rac1 antibody | Rabbit | WB: 1:1000 | GTX100761 | GeneTex |
| Phospho-Src Family (Tyr416) (D49G4) Rabbit mAb | Rabbit | WB: 1:1000  IHC:1:100 | 6943 | Cell Signaling Technology, Inc. |
| Src | Rabbit | WB: 1:500 | ARG65626 | Arigo |
| Anti-FAK (phospho Y397) antibody | Rabbit | WB: 1:1000  IF: 1:100 | ab81298 | Abcam |
| Fak | Rabbit | WB: 1:1000 | ab40794 | Abcam |
| GAPDH | Rabbit | WB: 1:4,000 | 10494-1-AP | ProteinTech Group, Inc. |
| ROCK2-Specific(C-Term) Antibody | Rabbit | IHC: 1:100 | 20248-1-AP | ProteinTech Group, Inc. |
| Transgelin antibody | Rabbit | WB: 1:1000;  IHC:1:100; IF:1:100 | GTX113561 | GeneTex |
| Phospho-Myosin Light Chain 2 (Ser19) Antibody | Rabbit | WB:1:1000  IF:1:50  IHC: 1:200 | 3671 | Cell Signaling Technology, Inc. |
| Alexa Fluor 488-conjugated goat secondary anti-rabbit IgGantibodies | Rabbit | IF: 1:200 |  | Antgene |
| Horseradish peroxidase (HRP)-conjugated goat anti-rabbit IgG | Goat | WB: 1:5,000 | ANT022 | Antgene |
| Horseradish peroxidase (HRP)-conjugated goat | Goat | WB: 1:5,000 | ANT19 | Antgene |

IHC, immunohistochemistry; WB, western blotting. IF: immunofluorescence

**Supplementary Table 2.** Details on the reagents used in the present study.

| **Reagents** | **Source** | **Identifier** |
| --- | --- | --- |
| Phalloidin–Tetramethylrhodamine B isothiocyanate | Sigma-Aldrich | P1951 |
| 3-amino-propionitrile | Sigma-Aldrich | CDS007521 |
| Dasatinib | MCE | HY-10181 |
| Blebbistatin | MCE | HY-13441 |
| Latrunculin A | Cayman | 76343-93-6 |
| Sulfo-SANPAH | Thermo | 22589 |
| RhoA activation assay kit | Cell Biolabs | STA-403-A |
| Collagen Type**Ⅰ** | MPbio | 150026 |
| Src Inhibitor 1 | MCE | HY-101053 |

**Supplementary Table 3. Individual Genes Used for Analysis of GSE30587 and GSE2109**

| COL1A2 |
| --- |
| COL2A1 |
| COL3A1 |
| COL4A1 |
| COL5A1 |
| COL11A1 |
| COL24A1 |
| COL27A1 |

**Supplementary Table 4. Patient characteristics used in Figure 1.**

|  | No. Age at diagnosis | FIGO stage Histologic type |  |
| --- | --- | --- | --- |
|  | 1 50  2 61  3 49  4 46  5 57  6 51  7 43  8　 46 | IIIC HGSOC  IIIC HGSOC  IIIC HGSOC  IIIC HGSOC  IIIC HGSOC  IIIC HGSOC  IIIC HGSOC  IIIC HGSOC |  |

Abbreviation: FIGO, International Federation of Gynecology and Obstetrics; HGSOC: High grade serous ovarian cancer.

**Supplementary Table 5. Patient characteristics related to Figure 3**

|  | Ovarian cancer (n=60) | Range of age (years) | 33-73 |
| --- | --- | --- | --- |
|  |  | **Histological type** |  |
|  |  | Serous Cystadenocarcinoma | 60 |
|  |  | **Tumor stage (FIGO)**  II  Ⅲ  Ⅳ | 5  43  12 |
|  |  | **Metastases**  Omentum metastases | 45 |
|  |  | Peritoneal metastases | 15 |

**Supplementary Table 6. Patient characteristics related to Figure 4**

|  | Ovarian cancer (n=132) | Range of age (years) | 27-77 |
| --- | --- | --- | --- |
|  |  | **Histological type** |  |
|  |  | Serous carcinoma | 94 |
|  |  | Mucinous carcinoma | 32 |
|  |  | Endometriod carcinoma | 5 |
|  |  | Clear cell carcinoma | 1 |
|  |  | **Tumor stage (FIGO)** |  |
|  |  | I | 13 |
|  |  | II | 7 |
|  |  | Ⅲ | 90 |
|  |  | Ⅳ | 22 |
|  |  |  |  |

**Supplementary Table 7. Patient characteristics related to Figure 5**

|  | Ovarian cancer (n=43) | Range of age (years) | 42-77 |
| --- | --- | --- | --- |
|  |  | **Histological type** |  |
|  |  | Serous carcinoma | 43 |
|  |  | **Tumor stage (FIGO)**  II  Ⅲ  Ⅳ | 2  34  7 |
|  |  |  |  |

**Supplementary Table 8. Patient characteristics related to supplementary figure 2**

|  | Ovarian cancer (n=16) | Range of age (years) | 42-61 |
| --- | --- | --- | --- |
|  |  | **Histological type** |  |
|  |  | Serous carcinoma | 16 |
|  |  | **Tumor stage (FIGO)** |  |
|  |  | Ⅲ  Ⅳ | 13  3 |
|  |  |  |  |
